# π–π Stacking Determines the Selectivity of Unnatural DNA Base Pairs Even without Polymerase

**DOI:** 10.1021/acsphyschemau.5c00100

**Published:** 2025-12-04

**Authors:** Zahra Noori, Andreu Bermejo, Josep Maria Bofill, Jordi Poater

**Affiliations:** † Departament de Química Inorgànica i Orgànica & IQTCUB, 16724 Universitat de Barcelona, Martí i Franquès 1-11, 08028 Barcelona, Spain; ‡ ICREA, Passeig Lluís Companys 23, 08010 Barcelona, Spain

**Keywords:** DFT, DNA replication, selectivity, stacking, unnatural DNA base pair

## Abstract

Expanding the genetic alphabet requires a mechanistic understanding of how synthetic bases are faithfully replicated alongside natural DNA. We present a quantum chemical study reproducing the experimentally observed single-nucleotide incorporation selectivity of Hirao’s unnatural base pairs (UBPs) by the 3′–5′ exonuclease-deficient Klenow fragment of *Escherichia coli* DNA polymerase I. Our analysis focuses on the highly selective DsPx pair, benchmarking its behavior against canonical Watson–Crick pairs and other UBPs. Strikingly, the observed selectivity emerges without explicitly modeling the polymerase, relying solely on computed stacking energies within the DNA helix. Molecular orbital and energy-decomposition analyses show that both electrostatic and dispersion interactions strengthen DsPx’s affinity more, capturing experimental fidelity trends and explaining its superior performance relative to related systems. We further evaluate other selective UBPs, including QPa, DsPa, and DsPn. Together, these results provide a quantitative framework for UBP incorporation selectivity and highlight the crucial role of noncovalent interactions in stabilizing synthetic bases within DNA. By bridging computation and experiment, this work advances design principles for synthetic genetic systems and contributes to unraveling the molecular origins of DNA replication fidelity.

## Graphical abstract

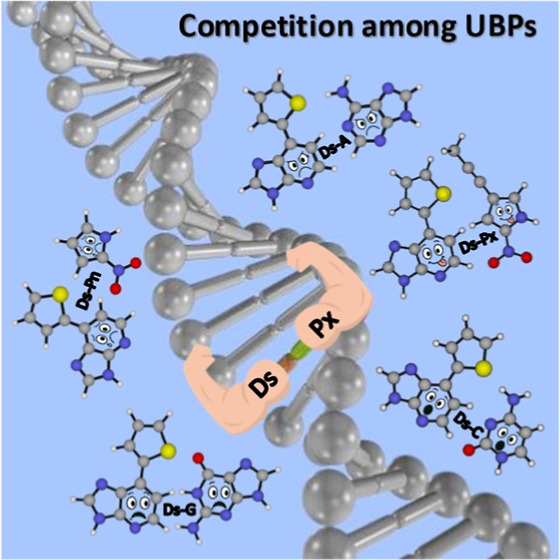

## 1 Introduction

DNA aptamers are short, single-stranded DNA molecules that bind specifically to a variety of targets, including small molecules, proteins, and cells.^1,2^ They are typically generated through an evolutionary process known as SELEX (Systematic Evolution of Ligands by EXponential enrichment), which involves repeated cycles of selection and polymerase chain reaction amplification (PCR) using DNA libraries with randomized sequences.^3^ Once the aptamer sequences are identified via SELEX, they are synthesized chemically, enabling the production of highly pure DNA aptamers. This method allows for precise sequence control and site-specific chemical modifications, making these newly synthesized DNA aptamers attractive alternatives to antibodies due to their reproducibility and consistency.

Compared to protein-based antibodies, DNA aptamers are inherently more hydrophilic and highly soluble in water, which helps avoid the nonspecific “stickiness” often associated with antibodies.^3,4^ However, this hydrophilic nature also weakens the hydrophobic interactions essential for strong binding to many protein targets. As a result, conventional aptamers composed solely of the four natural nucleotides—adenine (A), guanine (G), cytosine (C), and thymine (T)—often exhibit insufficient affinity for practical applications.^5–7^ To address this limitation, several chemical modification strategies have been explored.^8–10^ One common approach involves attaching hydrophobic groups to the natural DNA bases to enhance target binding.^11–13^

As a novel strategy to improve binding affinity, a genetic alphabet expansion method was developed by Hirao and co-workers by introducing a highly hydrophobic unnatural base (UB)—7-(2-thienyl)­imidazo­[4,5-*b*]­pyridine (Ds)—as a fifth nucleotide.^14^ This base specifically pairs with a modified partner, 2-nitro-4-propynylpyrrole (Px), which is functionalized with a diol group (Figure 1). The Ds–Px pair forms a third base pair that can be replicated with high fidelity during PCR amplification.^15,16^ Using this expanded base pair system, the authors developed a modified SELEX technique called ExSELEX (genetic alphabet expansion SELEX), which utilizes DNA libraries incorporating the Ds base. This method successfully yielded XenoAptamers—Ds-containing aptamers with markedly enhanced affinities for protein targets.^2,17,18^

**Figure 1:**
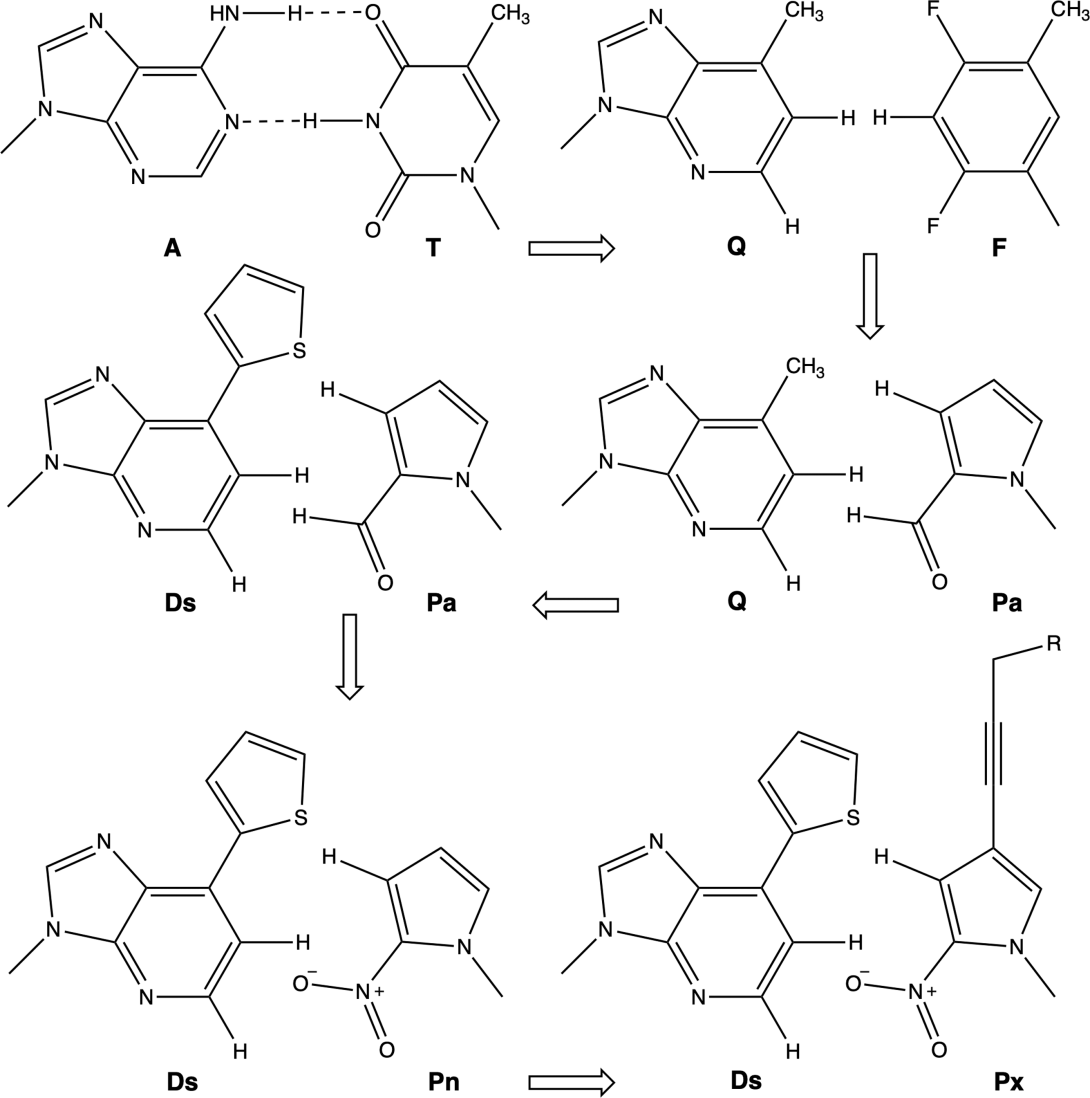
Hirao’s DsPx UB pair development process.

However, where does DsPx come from? In the mid-1990s, Morales and Kool developed hydrophobic analogs of the AT Watson–Crick base pair. In particular, non-hydrogen bonding UB pairs (UBPs), like QF and ZF structures depicted in Figure 1, are capable of DNA replication.^19,20^ Next, improvements by Hirao and co-workers led to the QPa pair using pyrrole-2-carboxaldehyde (Pa), which showed better shape complementarity and replication selectivity than QF.^21^ To enhance selectivity and avoid mispairing (e.g., Q with T), the same team developed a new hydrophobic base, Ds, which paired with Pa.^22^ They used γ-amidotriphosphates to suppress mispairing (either DsDs or APa), achieving the high-fidelity PCR amplification of DsPa pairs (99.9% selectivity after 20 cycles). To eliminate reliance on γ-amidotriphosphates, they created improved Pa analogs. Replacing the aldehyde with a nitro group yielded Pn, reducing mispairing with A. And finally, further enhancement led to Px (2-nitro-4-propynylpyrrole), which offered increased hydrophobicity and polymerase interaction, as pointed out above.^1^ In addition, the added propynyl group causes an increase of the stacking with adjacent base pairs, also avoiding DsDs mispairing.

In earlier work by our group, we showed that DNA replication depends critically on π–π stacking interactions and aqueous solvation.^23–26^ We specifically highlighted the interplay among hydrogen bonding, solvation, twist angles, and π–π stacking within a template–primer complex and demonstrated how these factors govern the stability of both complementary and mismatched base pairs. Building on that foundation, the study examined the stability of Watson–Crick and UB pairs, as well as the intrinsic ability of the template–primer complex to select the correct complementary base in the absence of DNA polymerase.^23^ To achieve this, we designed DNA model systems that operate independently of the polymerase active site, leveraging the fact that template-assisted chemical primer extension can reach base selectivity levels of up to 97% without enzymatic support.^27,28^ This strategy allowed us to isolate and probe the detailed interactions between DNA bases without interference from amino acid residues in the active site.

Now, our goal is to reproduce, using quantum chemical methods, the single-nucleotide incorporation efficiency and selectivity of various UB pairs (UBPs) developed by Hirao,^1^ as observed with the 3′–5′ exonuclease-deficient Klenow fragment of *Escherichia coli* DNA polymerase I. In particular, we focus on those UBPs with the highest reported selectivity—namely, QPa, DsPa, DsPn, and DsPx.^2,15,16^ We aim to be able to explain, through quantum chemistry, the higher selectivity of DsPx as depicted in Figure 4 of the referred work by Hirao and co-workers.^1^ By using known DNA bases alongside the proposed UB pair DsPx as a starting point, our approach aims to deepen the understanding of how UBPs can be incorporated into DNA helix and the corresponding results can be used in further studies, with emphasis on those on DNA replication.

## 2 Computational Methods

All calculations were carried out with the Amsterdam Density Functional (ADF) program using dispersion-corrected density functional theory at the ZORA-BLYP-D3­(BJ)/TZ2P level of theory (Table S3).^29–34^ The chosen functional has been demonstrated to reliably reproduce hydrogen-bonding geometries and energies for A–T and G–C Watson–Crick base pairs as well as stacked nucleotide arrangements. It also provides accurate interaction and bonding energies for π–π-stacked base systems. Both π-stacking and hydrogen-bonding energetic values obtained with this method closely match high-level CCSD­(T) benchmark results.^35,36^ To mimic the near-planar structure of B-DNA, isolated bases and hydrogen-bonded base pairs were optimized under a *C*_*s*_ symmetry constraint. Prior studies have shown that fully optimized *C*_*s*_-symmetric planar structures and *C*_1_-symmetric nonplanar structures exhibit virtually identical stabilities. The difference in bond energies between relaxed *C*_1_ and *C*_*s*_ conformations is generally within 0.1 kcal mol^–1^, except for the G–A system, which deviates by 0.6 kcal mol^–1^ due to amino-group pyramidalization.^23,37^

The effect of solvation in water was simulated by means of the Conductor-like Screening Model of solvation as implemented in ADF. The through-space interaction was analyzed within the framework of quantitative Kohn–Sham molecular orbital theory in combination with a quantitative energy decomposition analysis (EDA) in the gas phase. The interaction energy Δ*E*_int_ between the tetrazoles and aryl fragments was decomposed into the classical electrostatic attraction Δ*V*_elstat_, Pauli repulsion Δ*E*_Pauli_ between occupied orbitals, stabilizing orbital interactions Δ*E*_oi_, and dispersion Δ*E*_disp_.^38^ Finally, the electron density distribution is analyzed by using the Voronoi deformation density (VDD) method for atomic charge.^39^ Non-Covalent Interaction (NCI) plots have been computed by means of the Multiwfn software.^40,41^

### 2.1 Model System

To begin, each nucleotide base was fully optimized without symmetry constraints (*C*_*s*_ symmetry). We then evaluated the affinity between pairs of DNA bases, whether they form canonical Watson–Crick hydrogen bonds or represent non-natural base pairs that do not naturally hydrogen-bond. For this analysis, the individually optimized bases were positioned to favor potential hydrogen-bond formation, after which a geometry optimization was carried out while enforcing the *C*_*s*_ symmetry. The orientation of the hydrogen-bonded—or nonbonded—pairs, specifically the placement of the terminated glycosidic bond, was chosen to reproduce the experimentally observed α-helical twist of DNA.^23–26^ Then, the X–Y base pair bonding energy is computed as Δ*E* = *E*(X–Y) – *E*(X) – *E*(Y), being both the base pair and the bases alone fully optimized in *C*_*s*_ symmetry.

The second part of this study focuses on π-stacking interactions between base pairs. Stacked dimers were constructed using *C*_*s*_-optimized base pairs. To isolate stacking effects, the sugar–phosphate backbone was removed and replaced with hydrogen atoms, enabling us to study interactions directly between the nucleobases.^37,42,43^ The stacked arrangement was modeled by fixing the interpair separation at 3.4 Å and imposing a 36° twist angle, mimicking the influence of the backbone while preventing relaxation of the stacked structure (Figure 2). For this latter rotation, first the base pair is geometrically centered in the *xy* plane, with the two glycosidic bonds aligned in the *x*-axis, and next, the 36° rotation is applied in the *z*-axis around this center. This twist angle is between the two planes arisen from the straight lines connecting these two added H atoms that would give the glycosidic bond of each base. Noticeably, this simplified stacking model, which omits the backbone, has also been employed previously in studies of ring-expanded nucleobases. Prior work by Wetmore and co-workers demonstrated that although including the phosphodiester backbone can slightly alter the stationary points associated with nucleotide deglycosylation, backbone-truncated models with capped glycosidic linkages strike an effective balance between computational accuracy and efficiency.^44–46^ Nonetheless, with the aim to further support our model system, we have also performed the optimization of the DsPx/GC-stacked base pair dimer including the backbone together with two sodium atoms (Figure S4), proving the good agreement with our model system and supporting previous works undertaken with the same methodology. Finally, the X–Y/X′–Y′ stacking energy is computed as Δ*E* ^stacking^ = *E*(X–Y/X′–Y′) – *E*(X–Y) – *E*(X′–Y′), being the base pairs fully optimized in *C*_*s*_ symmetry, both alone and in the built stacked system. And for the stacked trimer, X″–Y″/X–Y/X′–Y′, its stacking energy is computed as Δ*E* ^stacking^ = *E*(X″–Y″/X–Y/X′–Y′) – *E*(X″–Y″) – *E*(X–Y) – *E*(X′–Y′).

**Figure 2:**
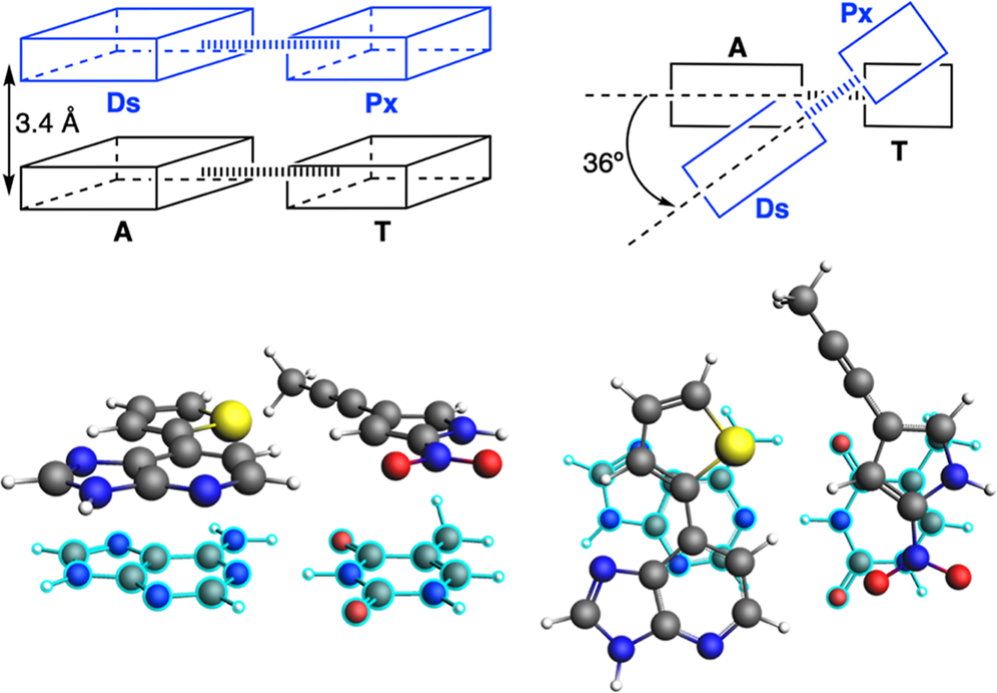
Schematic (top) and on-scale atomistic (bottom) representation of the complex of the DsPx base pair stacked to AT (colored in light blue) at a distance of 3.4 Å and with a mutual rotation of 36°. Front view (left) and top view (right) of the stacked base pair dimer.

## 3 Results and Discussion

The first step is to analyze the bonding energies of the set of base pairs. As a reference, Watson–Crick AT and GC base pairs present total bonding energies of −8.7 and −13.4 kcal mol^–1^, respectively, computed at the ZORA-BLYP-D3­(BJ)/TZ2P level of theory in water. The stronger GC is attributed to the presence of three hydrogen bonds, compared to two for AT (Figure 3 and Table S1).^23–26^ Noticeably, computed hydrogen bond lengths are very close to those present in Dickerson’s dodecamer.^47,48^ Next, we have considered recently designed UB pairs (UBPs), together with those formed between Ds or Q with the four DNA bases, with the aim to analyze their selectivity. At difference from WC base pairs, all UBPs under analysis present very weak bonding energies. For instance, in the case of Ds, the bonding energies of the base pairs amount between −2.6 and −1.0 kcal mol^–1^ (Figure 4), whereas in the case of Q, Δ*E* amounts between −1.0 and −0.2 kcal mol^–1^ (Figure 5). Such weaker bonding energies compared to WC energies must be attributed to the lack of hydrogen bonds in these UBPs. All interactions are much longer than 2.0 Å (Figures 4 and 5), thus supporting their weak nature. Importantly, all base pairs have been constrained to *C*_*s*_ symmetry to mimic the planarity in DNA helix, in which the base pairs have been previously proven to present an almost planar geometry.^49^ Nonetheless, we have checked that the maximum energy difference between constrained and fully relaxed bases amounts to be less than 0.2 kcal mol^–1^.

**Figure 3:**
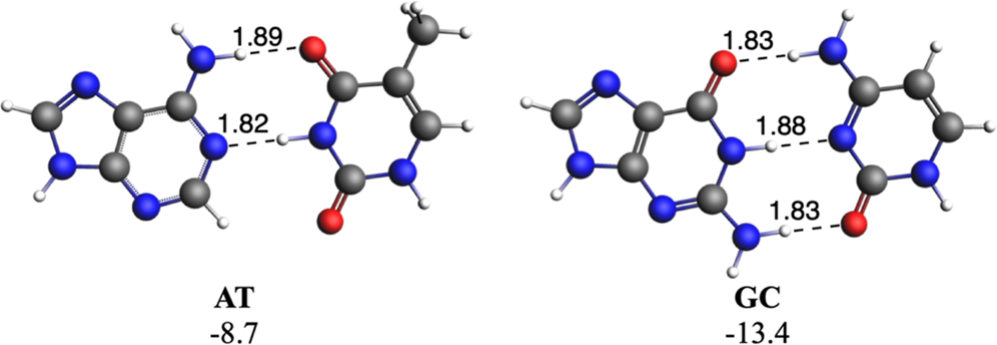
Watson–Crick AT and GC base pairs. Hydrogen bond lengths (in Å) and bonding energies (in kcal mol^–1^) are also enclosed. Computed at the ZORA-BLYP-D3­(BJ)/TZ2P level of theory in water.

**Figure 4:**
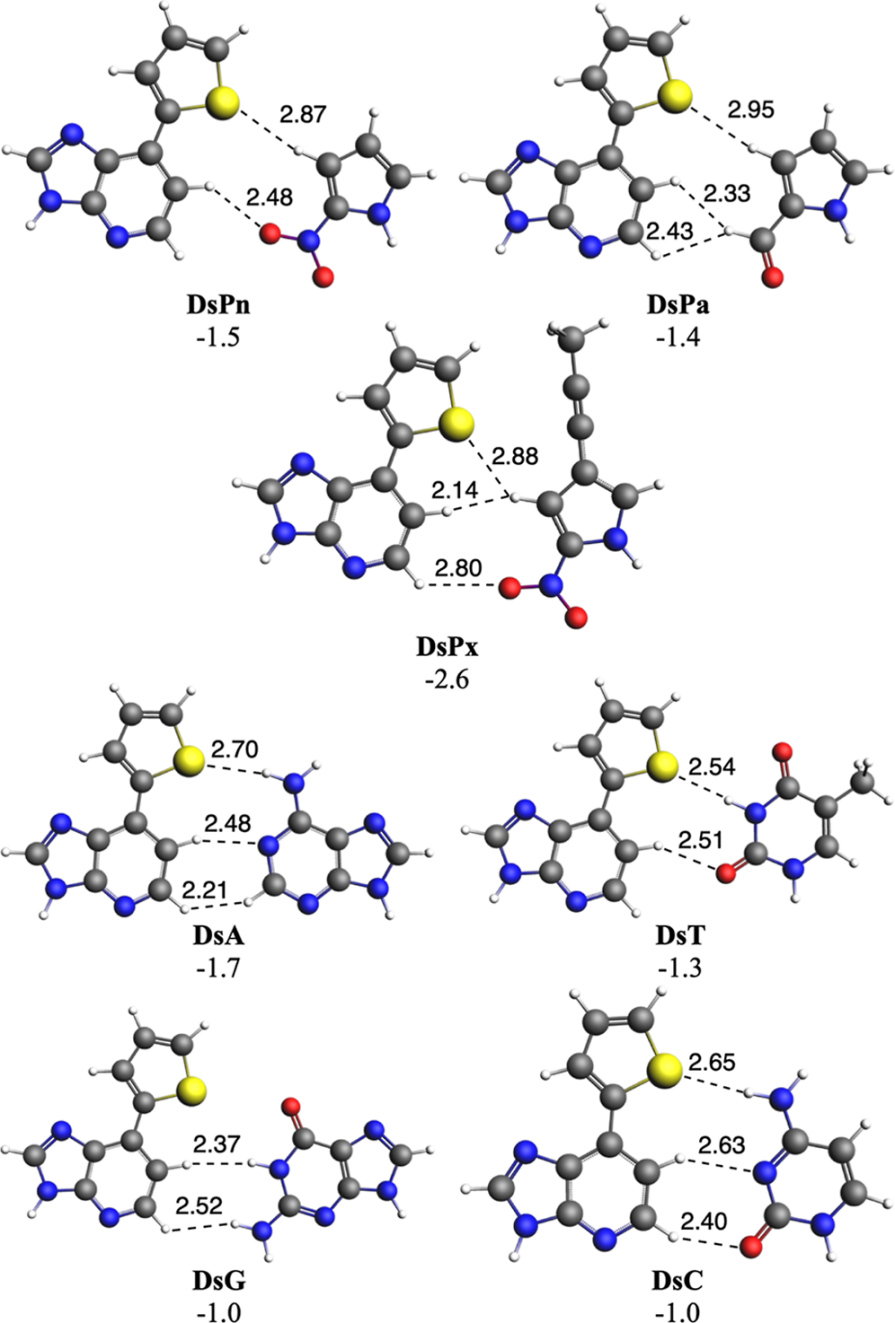
DNA base pairs with unnatural Ds. Main bond lengths (in Å) and bonding energies (in kcal mol^–1^) are also enclosed. Computed at the ZORA-BLYP-D3­(BJ)/TZ2P level of theory in water.

**Figure 5:**
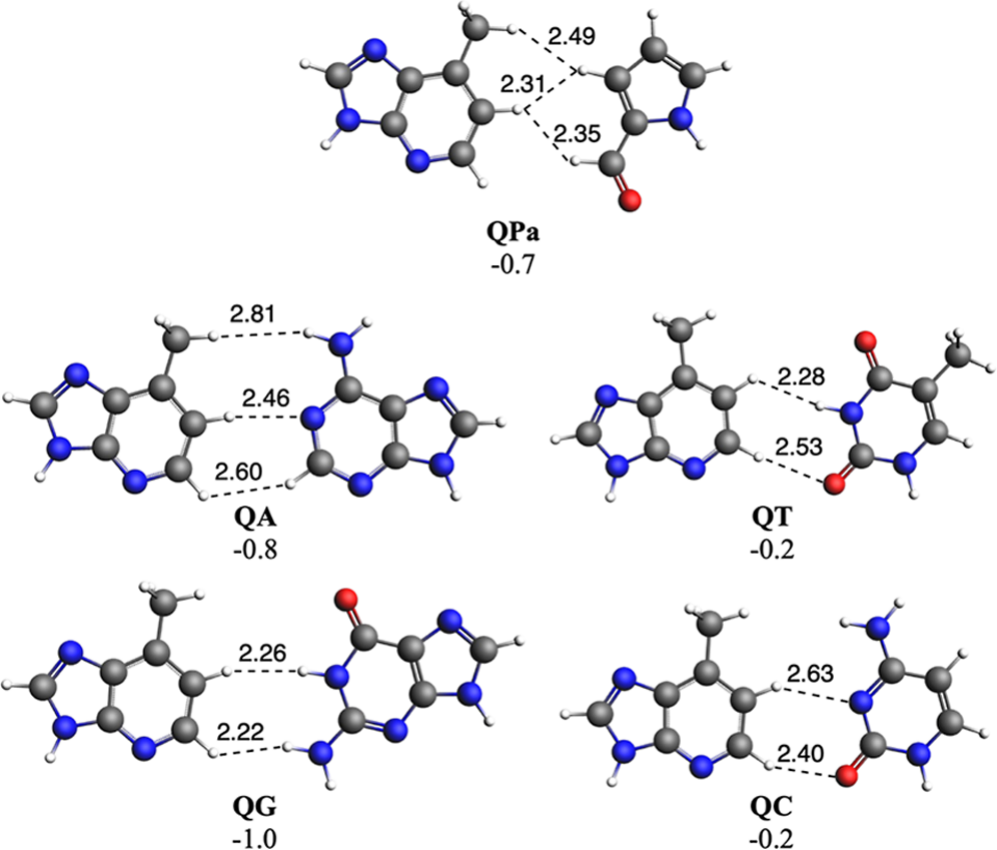
DNA base pairs with unnatural Q. Main bond lengths (in Å) and bonding energies (in kcal mol^–1^) are also enclosed. Computed at the ZORA-BLYP-D3­(BJ)/TZ2P level of theory in water.

The aim of this analysis is to check the selectivity of these newly proposed UBPs, i.e., how favorable are either DsPn, DsPa, or DsPx compared to Ds binding any of the DNA bases (DsA, DsC, DsG, or DsT). For such an aim, a model system was built without polymerase (see the Computational Details section). The stacked systems were prepared by separating the base pairs 3.4 Å, which is the average distance in DNA, and rotating them to a twist angle of 36°, which corresponds to the average twist in B-DNA (Figure 2). In all the systems studied, the backbone was removed, hydrogen atoms have been inserted in its position, and a *C*_*s*_ symmetry was imposed to mimic the nearly planar arrangement observed in B-DNA.

Outstandingly, the UB pair DsPx appears to present the strongest interaction with either Watson–Crick AT or GC (Figure 6). Thus, the preferred selectivity of the formation of DsPx is also confirmed by its stacking strength to a natural base pair. In particular, DsPx presents stacking energies (Δ*E* ^stack^) that amount to −11.7 and −12.6 kcal mol^–1^ with either AT or GC, respectively. Its higher affinity is supported by the difference of 1.0 and 2.0 kcal mol^–1^ in Δ*E* ^stack^ compared to the possible formation of any other base pair with Ds. The preference for DsPx is followed by DsPn (Δ*E* ^stack^ = −10.4 and −10.6 kcal mol^–1^ with AT and GC, respectively), the UBP from which it was derived, thus also supporting its good performance compared to the previously developed ones. However, its Δ*E* ^stack^ is closely followed by other possible base pairs. For instance, DsPa follows next (Δ*E* ^stack^ = −10.3 and −10.3 kcal mol^–1^ with AT and GC, respectively), although it is not being preferred compared to its binding with natural bases. Also noticeably, the stacking energies with either AT or GC present quite similar values, with a maximum difference of 0.82 kcal mol^–1^ in the case of the most favored DsPx when stacked to GC. At this point, it must be pointed out that, despite the fact that we have referred to this preference for DsPx as selectivity, the term affinity is better suited based on the small energy difference.

**Figure 6:**
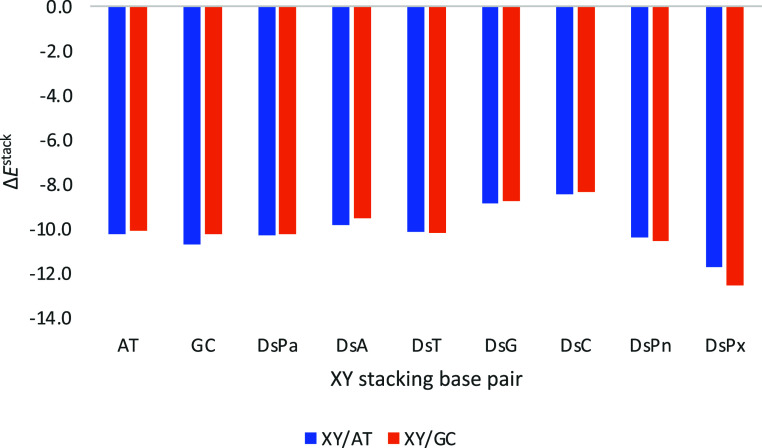
Stacking interaction (in kcal mol^–1^) of the considered base pairs (XY) with Ds, and Watson–Crick for comparison, on top of AT or GC base pairs with a twist angle of 36°. Computed at the ZORA-BLYP-D3­(BJ)/TZ2P level of theory in water.

If the bottom base pair is reversed, i.e., instead of AT or GC, we put TA or CG, the trends are kept (Figure 7). DsPx interacts the strongest (Δ*E* ^stack^ = −13.0 and −12.7 kcal mol^–1^ with TA and CG, respectively). The unnatural DsPn (Δ*E* ^stack^ = −12.0 and −11.9 kcal mol^–1^ with TA and CG, respectively) and DsPa (Δ*E* ^stack^ = −12.2 and −11.7 kcal mol^–1^ with TA and CG, respectively) follow next. Noticeably, in this case, the formation of UBPs presents a clear preference compared to Ds binding any of the four natural DNA bases.

**Figure 7:**
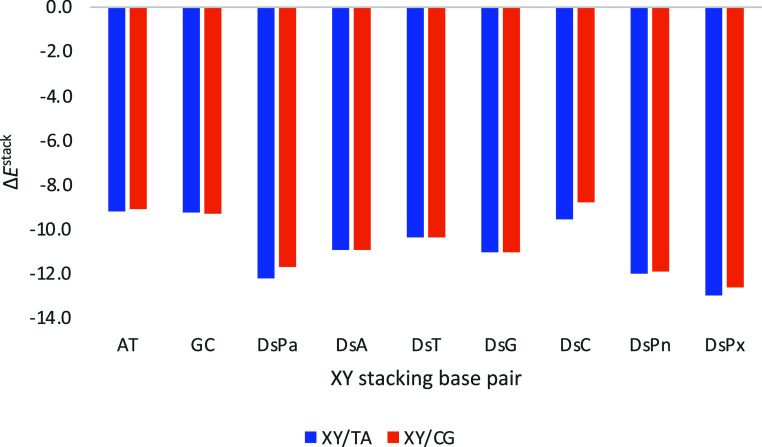
Stacking interaction (in kcal mol^–1^) of the considered base pairs (XY) with Ds, and Watson–Crick for comparison, on top of TA or CG base pairs with a twist angle of 36°. Computed at the ZORA-BLYP-D3­(BJ)/TZ2P level of theory in water.

At difference, when the UBPs are stacked at the bottom of either AT or GC, DsPx is not the preferred one, but other base pairs stack stronger (Figure S1). In particular, DsA presents a stronger stacking energy (−14.4 and −13.5 kcal mol^–1^ with AT and GC, respectively) than that of DsPx (−12.5 and −12.4 kcal mol^–1^ with AT and GC, respectively). However, this is not the case with reversed TA or CG (Figure S2), when again DsPx presents the strongest stacking interaction (−13.9 and −13.1 kcal mol^–1^ with AT and GC, respectively), followed by DsA (−13.3 and −12.4 kcal mol^–1^ with AT and GC, respectively). Thus, based on a model system with two stacked base pairs, being one the UBP and the second a Watson–Crick one, in almost all cases, the preferred affinity of DsPx is determined by its stronger stacking interaction with the Watson–Crick base pair. This is not the case with either previously designed DsPa or DsPn that, despite also presenting a relatively similar Δ*E* ^stack^ to DsPx, values are very close or even smaller to those base pairs of Ds binding natural DNA bases.

The same analysis above has been applied to QPa, with the aim to check its selectivity compared to Q forming either QA, QC, QG, or QT (Figure 8). QPa presents the strongest stacking energy with either AT or GC (−9.7 and −9.9 kcal mol^–1^ with AT and GC, respectively). Thus, QPa, which was even designed before DsPx, is preferred over Q binding to any of the natural bases just based on the stacking interaction with Watson–Crick DNA bases.

**Figure 8:**
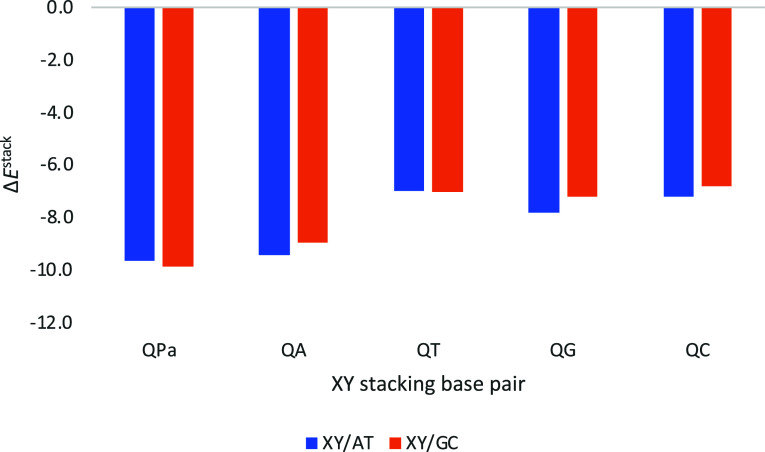
Stacking interaction (in kcal mol^–1^) of the considered base pairs (XY) with Q, and Watson–Crick for comparison, on top of AT or GC base pairs with a twist angle of 36°. Computed at the ZORA-BLYP-D3­(BJ)/TZ2P level of theory in water.

With the aim to get closer to a more realistic DNA helix, we placed the UBPs stacked between two Watson–Crick base pairs (Figure 9). As the dimer model system above, each base pair is twisted 36° with respect to the next one.

**Figure 9:**
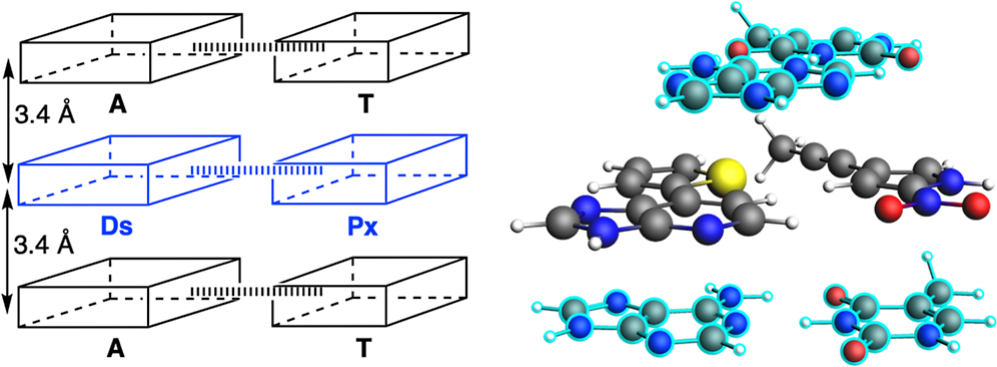
Schematic (left) and on-scale atomistic (right) representation of the complex of DsPx base pair stacked between two AT (colored in light blue) base pairs at a distance of 3.4 Å and with a mutual rotation of 36°.

DsPx once again appears as the UPB that presents the strongest stacking interaction. Whatever the combination of AT and GC is, DsPx stacks stronger (between −24.7 and −25.6 kcal mol^–1^) than any of the other UBPs (Figure 10). Both previously designed DsPa and DsPn also present strong Δ*E* ^stack^ energies (between −22.6 and −23.4 kcal mol^–1^). These latter are only weaker than those by DsA (between −23.5 and −24.7 kcal mol^–1^).

**Figure 10:**
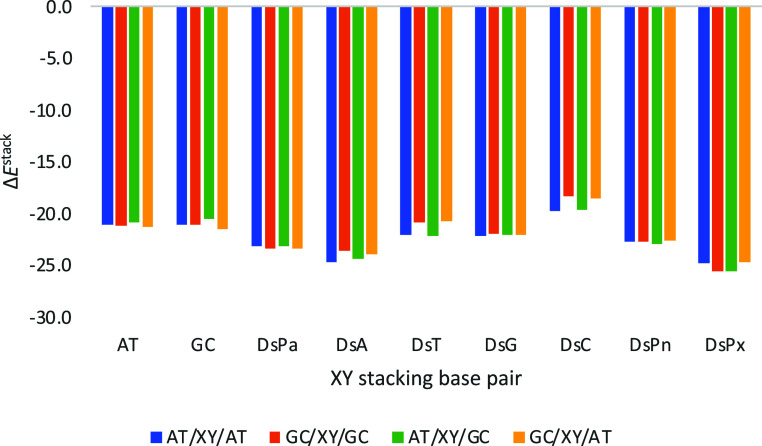
Stacking interaction (in kcal mol^–1^) of the considered base pairs with Ds, located in between Watson–Crick base pairs forming a trimer with a twist angle of 36°. Computed at the ZORA-BLYP-D3­(BJ)/TZ2P level of theory in water.

Noticeably, DsPx is also preferred if it is reversed, i.e., PxDs, and also placed in the same trimer model system (Figure 11). Δ*E* ^stack^ energies are even larger compared to the original orientation (from −26.3 to −27.4 kcal mol^–1^). Then, DsPa directly competes with DsA, closely followed by DsPn.

**Figure 11:**
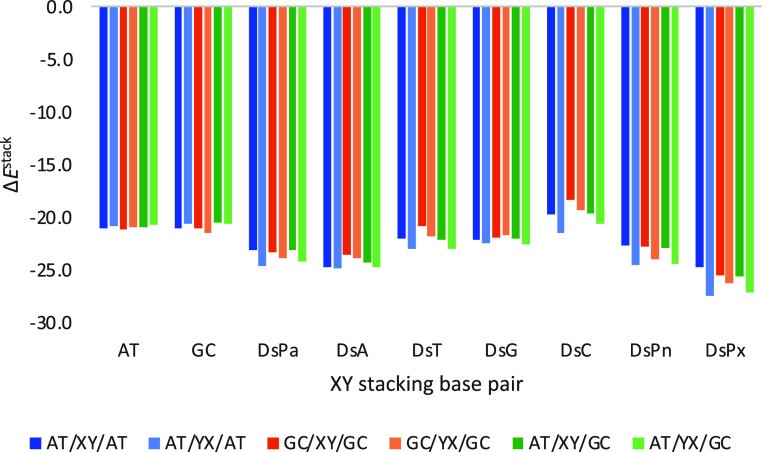
Stacking interaction (in kcal mol^–1^) of the considered base pairs with Ds, located in between Watson–Crick base pairs forming a trimer with a twist angle of 36°. For nomenclature, whereas XY refers to DsPx, YX refers to PxDs. Computed at the ZORA-BLYP-D3­(BJ)/TZ2P level of theory in water.

Overall, with our model system, we are able to reproduce the observed experimental single-nucleotide incorporation selectivity of Hirao’s UB pairs by the 3,5-exonucleobase-deficient Klenow fragment of *E. coli* DNA polymerase I.^1,2^ Noticeably, our model system does not include polymerase; thus, such selectivity is exclusively already reproduced by means of computed stacking energies in the DNA helix, giving rise to stronger affinities.

However, can we obtain further insight regarding the reason for this stronger affinity by the UBP DsPx compared to that of either Watson–Crick AT or GC? Why is such enhanced stacking of DsPx responsible for its better selectivity among the other possible formed base pairs? To address this issue, a quantitative Kohn–Sham molecular orbital analysis^50^ together with an EDA has been performed on two extreme cases discussed above, i.e., DsPx and AT, and each of them stacked on top of both AT and GC.

First, even without the aqueous environment, DsPx stacks stronger than AT on top of either AT (−14.9 vs −14.0 kcal mol^–1^) or GC (−16.0 vs −14.1 kcal mol^–1^, Table 1). These values are larger than those in water due to the effect of the solvation energy in each base pair compared to the whole dimer.^23–25^ Let us first focus on the dimers stacked with GC. Despite the more unfavorable Pauli repulsion DsPx experiences (20.6 vs 16.6 kcal mol^–1^), it is compensated by more attractive electrostatic (−7.1 vs −4.9 kcal mol^–1^) and dispersion (−25.2 vs −21.9 kcal mol^–1^) interactions and also by slightly more favorable orbital interactions (−4.3 vs −3.8 kcal mol^–1^), thus giving rise to an enhanced stacking interaction compared to AT (Table 1). We can go further by analyzing the individual interactions in DsPx/GC, i.e., between Ds and G, and between Px and C, and also their cross terms (Table 2), and compare to those for the AT/GC-stacked dimer. Noticeably, the cross interactions are the ones responsible for the stronger stacking of DsPx compared to AT with GC. In particular, Px/G and Ds/C are stronger than T/G and A/C by 1.6 and 3.8 kcal mol^–1^, respectively. And the reason is found in the more favorable electrostatic interactions between these involved bases (by −1.6 and −3.7 kcal mol^–1^, respectively) but also the more attractive dispersion interaction in the case of Ds/C compared to A/C (by −3.0 kcal mol^–1^, Table 2). The more favorable, i.e., more attractive, electrostatic interaction in the case of DsPx than AT stacked to GC comes from their different electronic structure, as supported by the VDD charges distribution (Figure 12), as well as by their molecular electrostatic potential (MEP) isosurfaces (Figures 13 and S3).

**Table 1:** EDA (in kcal mol^–1^) of the Stacking Interaction of the DsPx and AT Base Pairs on Top of the AT or GC Base Pairs with a Twist Angle of 36°^a^

	Δ*E*_int_	Δ*E*_Pauli_	Δ*V*_elstat_	Δ*E*_oi_	Δ*E*_disp_
DsPx/AT	–14.9	23.1	–7.8	–4.4	–25.8
AT/AT	–14.0	17.3	–5.8	–3.4	–22.1
DsPx/GC	–16.0	20.6	–7.1	–4.3	–25.2
AT/GC	–14.1	16.6	–4.9	–3.8	–21.9

a Computed at the ZORA-BLYP-D3­(BJ)/TZ2P level of theory in gas on the geometries of the base pairs computed in water. Δ*E*_int_ = Δ*E*_Pauli_ + Δ*V*_elstat_ + Δ*E*_oi_ + Δ*E*_disp_.

**Table 2:** EDA (in kcal mol^–1^) of the Stacking Interaction of the DsPx and AT Base Pairs on Top of the GC Base Pair with a Twist Angle of 36°, together with the Individual Contributions of the Bases and the Cross Terms^a^

		Δ*E*_int_	Δ*E*_Pauli_	Δ*V*_elstat_	Δ*E*_oi_	Δ*E*_disp_
DsPx/GC	DsPx/GC	–16.0	20.6	–7.1	–4.3	–25.2
	Ds/G	–6.3	8.0	–2.3	–1.9	–10.1
	Px/C	–4.9	7.6	–2.2	–2.0	–8.3
	Px/G	–2.0	1.2	–0.7	–0.5	–2.0
	Ds/C	–3.7	4.6	–2.4	–1.0	–4.8
AT/GC	AT/GC	–14.1	16.6	–4.9	–3.8	–21.9
	A/G	–7.6	7.5	–3.6	–1.9	–9.6
	T/C	–6.5	7.8	–3.2	–2.4	–8.7
	T/G	–0.4	1.0	0.9	–0.4	–1.9
	A/C	0.1	1.1	1.2	–0.4	–1.8

a Computed at the ZORA-BLYP-D3­(BJ)/TZ2P level of theory in gas on the geometries of the base pairs computed in water. Δ*E*_int_ = Δ*E*_Pauli_ + Δ*V*_elstat_ + Δ*E*_oi_ + Δ*E*_disp_.

**Figure 12:**
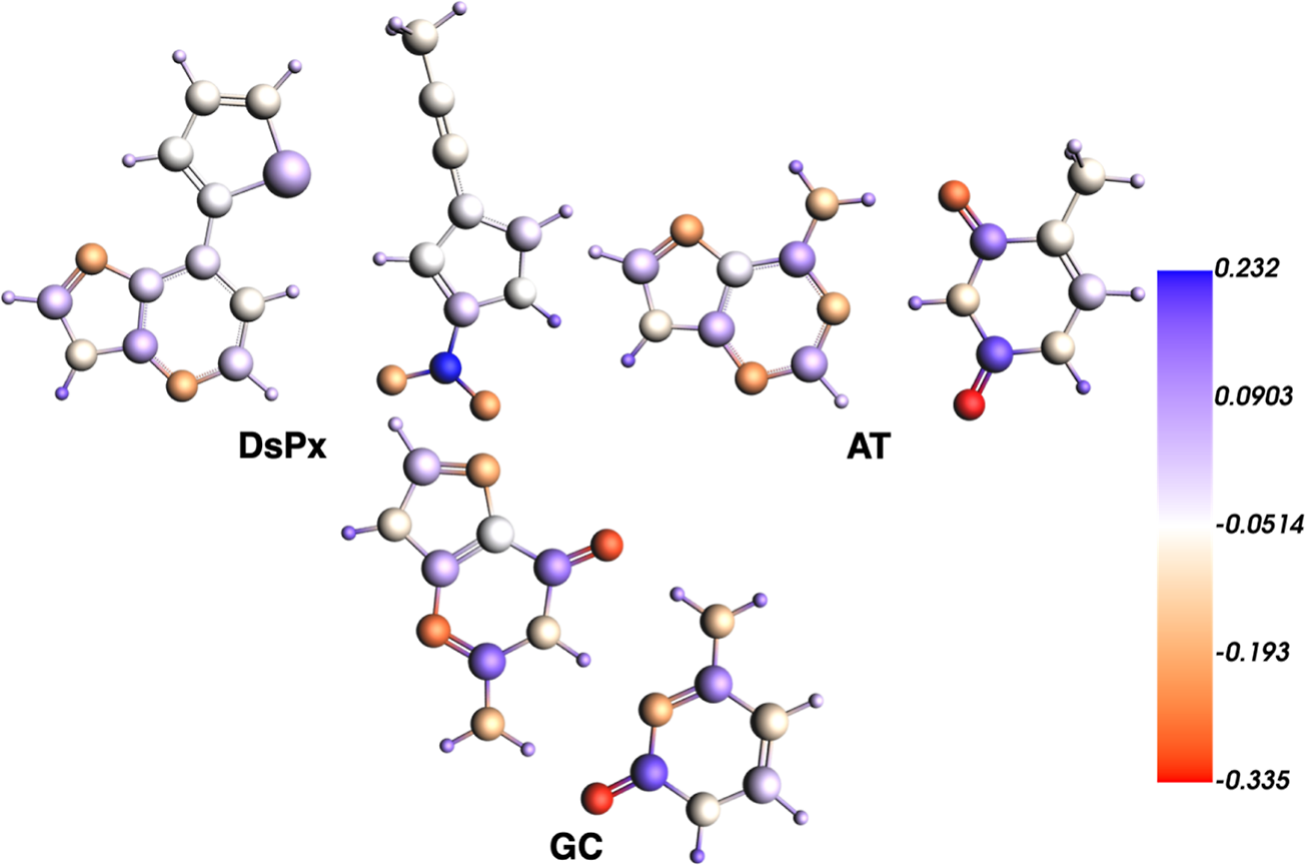
VDD charge isosurfaces (in e) of the base pairs involved in DsPx/GC and AT/GC dimers. GC is rotated with a twist angle of 36°.

**Figure 13:**
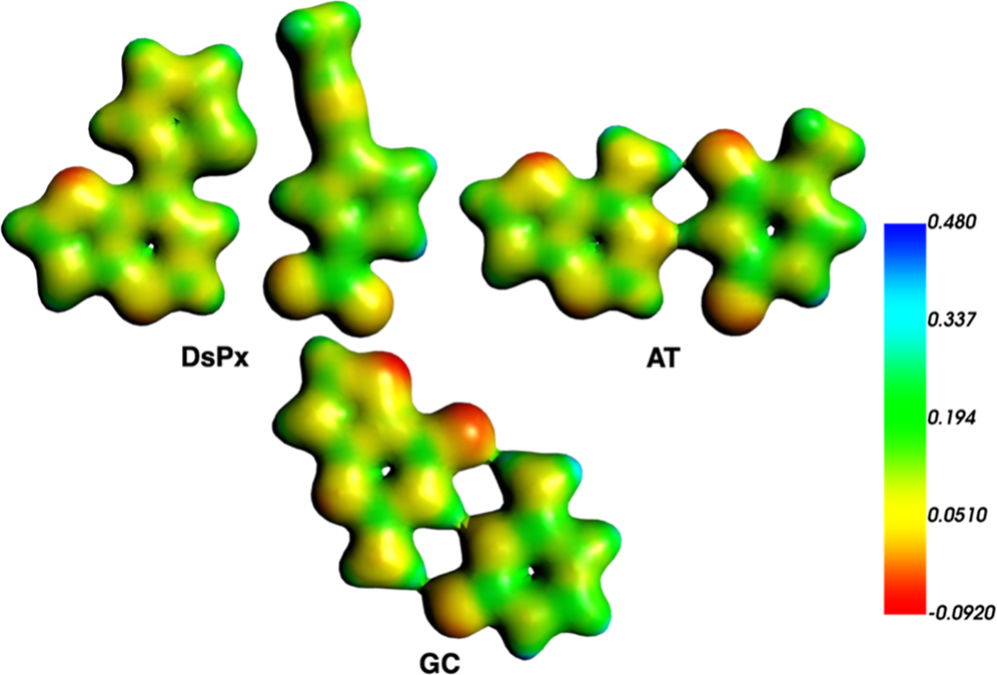
MEP isosurfaces (a.u., electronic density isovalue = 0.03 au) of the base pairs involved in DsPx/GC and AT/GC dimers. GC is rotated with a twist angle of 36°.

The above conclusions are not only restricted to these base pair dimers, but the same can be applied when comparing DsPx to AT stacked to AT (Table S2). In addition, the determinant role of both electrostatic and dispersion interactions is also applied to the rest of the possible UB pairs under analysis, whose values lie in between the two extreme cases we have analyzed in depth above.

Finally, and for completeness, we have also computed the NCI plots for both these latter dimers with the aim to better understand the better dispersion interaction in the case of DsPx stacked to GC (Figure 14). In the case of the AT/GC dimer, A shows an important noncovalent interaction with G; however, that between T and C is quite weak. At difference, in DsPx/GC, with a stacked base, dimers show such a noncovalent interaction, with a special emphasis on the propynyl group attached in Px interacting with C below. Thus, such observation agrees with the stronger dispersion interaction of this latter dimer compared to the Watson–Crick one.

**Figure 14:**
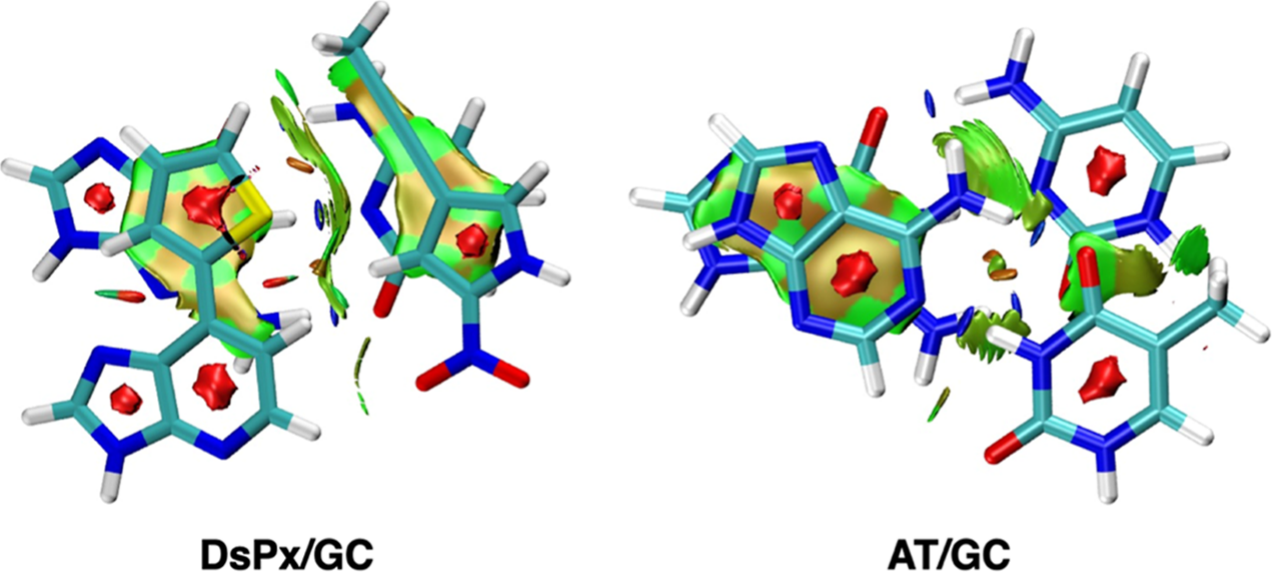
NCI plot of DsPx/GC and AT/GC dimers (promolecular density isovalue = 0.35 a.u.).

## 4 Conclusions

We have successfully reproduced, using quantum chemical methods, the experimentally observed single-nucleotide incorporation selectivity of Hirao’s UB pairs (UBPs) by the 3′–5′ exonuclease-deficient Klenow fragment of *E. coli* DNA polymerase I, originally developed by Hirao and co-workers.^1^ Our analysis focuses on the UBP that exhibits the highest selectivity, Ds–Px, and compares its performance not only with canonical Watson–Crick base pairs but also with other previously designed UBPs. Importantly, our model system excludes the polymerase itself, yet the experimentally observed selectivity emerges solely from the computed stacking energies within the DNA helix. Through quantitative molecular orbital and energy decomposition analyses, we demonstrate the decisive contributions of both electrostatic and dispersion interactions to the computed enhanced affinity of Ds–Px. Ultimately, our approach provides new insights into DNA replication—one of the most fundamental biological processes—whose molecular basis of remarkably high fidelity remains only partially understood.

## Supplementary Material



## Data Availability

The data underlying this study are available in the published article and its Supporting Information.

## Notes

Cartesian coordinates of all systems under analysis together with their ADF electronic energies and stacking interaction energies of the model systems (PDF)
