# Supplementary material for: π–π Stacking Determines the Selectivity of Unnatural DNA Base Pairs Even without Polymerase
Source: ACS Phys Chem Au. 2025 Dec 4;6(1):153–62. doi: 10.1021/acsphyschemau.5c00100 (PMC12856653; doi:10.1021/acsphyschemau.5c00100)
Supplement: Supplementary file 1 [file pg5c00100_si_001.pdf]

SUPPORTING INFORMATION

π-π Stacking Determines the Selectivity of Unnatural DNA Base Pairs even without Polymerase

Zahra Noori, Andreu Bermejo, Josep M. Bofill,* and Jordi Poater*

AUTHOR INFORMATION

Corresponding Authors

Jordi Poater - *Departament de Química Inorgànica i Orgànica & IQTCUB, Universitat de Barcelona, Martí i Franquès 1-11, 08028 Barcelona, Spain; ICREA, Passeig Lluís Companys 23, 08010 Barcelona, Spain; orcid.org/0000-0002-0814-5074;*

E-mail: jordi.poater@ub.edu

Josep Maria Bofill - *Departament de Química Inorgànica i Orgànica & IQTCUB, Universitat de Barcelona, Martí i Franquès 1-11, 08028 Barcelona, Spain; orcid.org/0000-0002-0974-4618;*

E-mail: jmbofill@ub.edu

Authors

Zahra Noori - *Departament de Química Inorgànica i Orgànica & IQTCUB, Universitat de Barcelona, Martí i Franquès 1-11, 08028 Barcelona, Spain*

Andreu Bermejo - *Departament de Química Inorgànica i Orgànica & IQTCUB, Universitat de Barcelona, Martí i Franquès 1-11, 08028 Barcelona, Spain*

Figure S1. Stacking interaction (in kcal mol^{-1}) of the considered base pairs with Ds on the bottom of AT or GC base pairs with a twist angle of 36º.

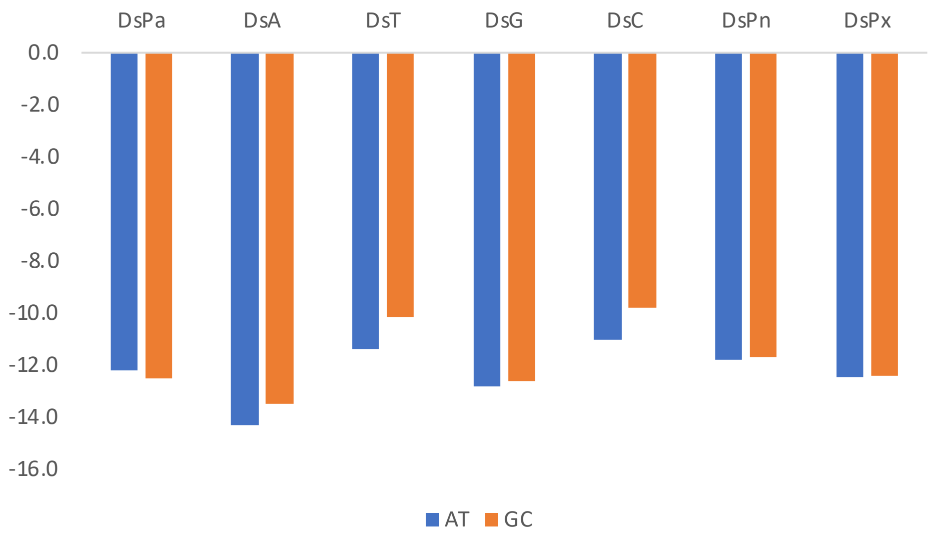

Figure S2. Stacking interaction (in kcal mol^{-1}) of the considered base pairs with Ds on the bottom of TC or CG base pairs with a twist angle of 36º.

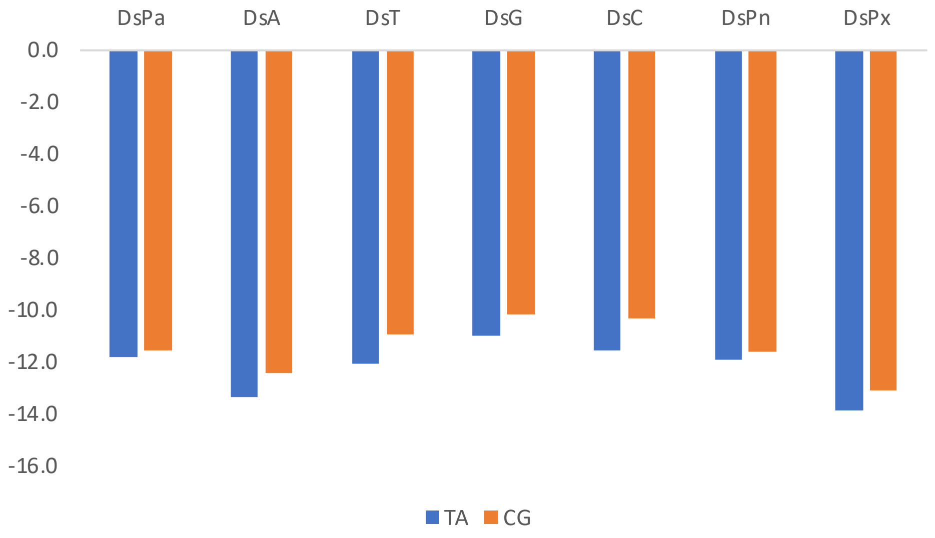

Figure S3. a) Molecular electrostatic potential isosurfaces (a.u., electronic density isovalue = 0.03 a.u.), and b) Voronoi deformation density (VDD) charges (in milli-e.) of the base pairs involved in DsPx/GC and AT/GC dimers.

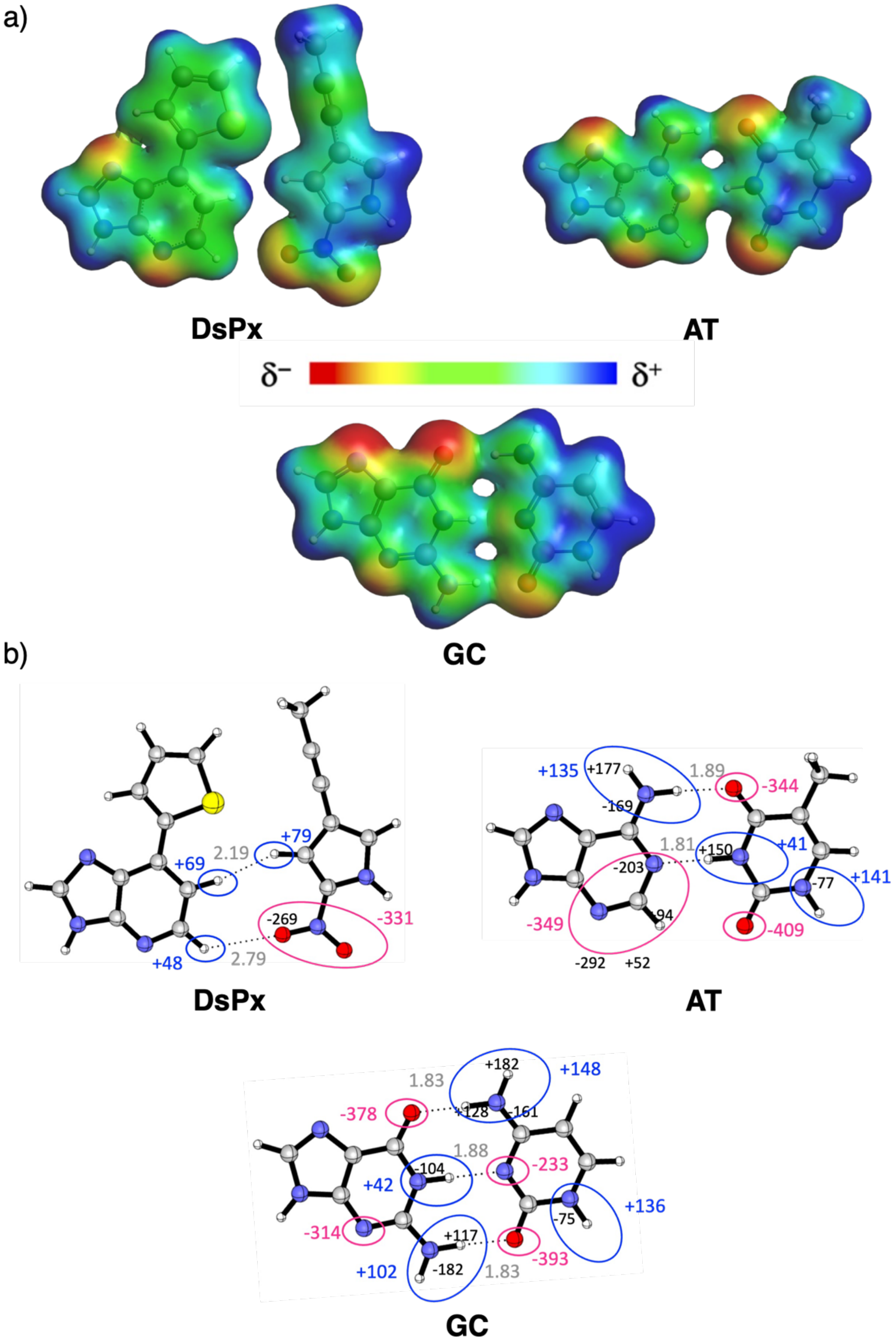

Figure S4. Geometry of stacked DsPx/GC system with backbone and two sodium atoms. Only planarity of the two base pairs has been constrained. For comparison, our model system of the same stacked complex has been added (underlined in blue). Computed at ZORA-BLYP-D3(BJ)/TZ2P in water.

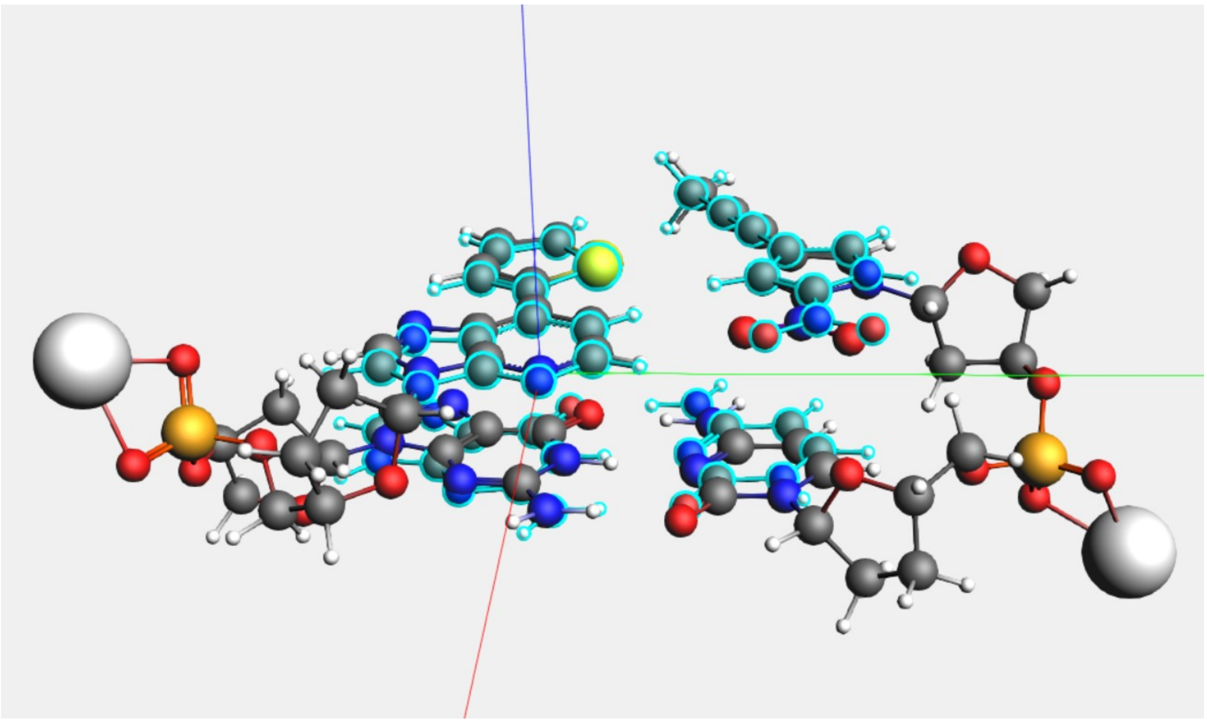

Table S1. Base pair bonding energies and stacking energies of the dimer and trimer model systems (in kcal mol^{-1}). Computed at ZORA-BLYP-D3(BJ)/TZ2P in water.

**Base Pair**	ΔE
AT	-8.66
GC	-13.36
DsPa	-1.44
DsA	-1.66
DsT	-1.30
DsG	-0.99
DsC	-1.04
DsPn	-1.53
DsPx	-2.62
QPa	-0.70
QA	-0.76
QT	-0.19
QG	-1.02
QC	-0.22

**Base Pair**	ΔE^{stack}	ΔE^{stack}
	**AT**	**GC**
AT	-10.2	-10.1
GC	-10.7	-10.2
DsPa	-10.3	-10.3
DsA	-9.8	-9.5
DsT	-10.1	-10.2
DsG	-8.9	-8.8
DsC	-8.4	-8.3
DsPn	-10.4	-10.6
DsPx	-11.7	-12.6

**Base Pair**	ΔE^{stack}	ΔE^{stack}
	**AT**	**GC**
TA	-9.2	-9.1

CG	-9.3	-9.3
PaDs	-12.2	-11.7
ADs	-10.9	-10.9
TDs	-10.4	-10.4
GDs	-11.0	-11.1
CDs	-9.6	-8.8
PnDs	-12.0	-11.9
PxDs	-13.0	-12.7

**Base Pair**	**Δ*E*^{stack}**	**Δ*E*^{stack}**
	**AT**	**GC**
DsPa	-12.3	-12.5
DsA	-14.4	-13.5
DsT	-11.4	-10.2
DsG	-12.8	-12.7
DsC	-11.0	-9.8
DsPn	-11.8	-11.7
DsPx	-12.5	-12.4

**Base Pair**	**ΔE^{stack}**	**ΔE^{stack}**
	**AT**	**GC**
PaDs	-11.8	-11.5
ADs	-13.3	-12.4
TDs	-12.1	-10.9
GDs	-11.0	-10.2
CDs	-11.5	-10.3
PnDs	-11.9	-11.6
PxDs	-13.9	-13.1

**Base Pair**	**ΔE^{stack}**	**ΔE^{stack}**
	**AT**	**GC**
QPa	-9.7	-9.9
QA	-9.4	-9.0

QT	-7.0	-7.0
QG	-7.8	-7.2
QC	-7.2	-6.8

**Base Pair**	ΔE^{stack}	ΔE^{stack}	ΔE^{stack}	ΔE^{stack}	ΔE^{stack}	ΔE^{stack}
	**AT/XY/AT**	**AT/YX/AT**	**GC/XY/GC**	**GC/YX/GC**	**AT/XY/GC**	**AT/YX/GC**
**AT**	-21.0	-20.8	-21.2	-21.0	-20.9	-20.7
**GC**	-21.1	-20.6	-21.0	-21.5	-20.5	-20.7
**DsPa**	-23.1	-24.7	-23.4	-23.9	-23.1	-24.2
**DsA**	-24.7	-24.8	-23.5	-23.9	-24.4	-24.8
**DsT**	-22.0	-23.0	-20.8	-21.8	-22.1	-23.0
**DsG**	-22.1	-22.5	-21.9	-21.7	-22.0	-22.5
**DsC**	-19.8	-21.5	-18.3	-19.3	-19.6	-20.6
**DsPn**	-22.7	-24.5	-22.8	-24.0	-22.9	-24.4
**DsPx**	-24.8	-27.4	-25.5	-26.3	-25.6	-27.1

Table S2. Energy decomposition analysis (in kcal mol^{-1}) of the stacking interaction of the DsPx and AT base pairs on top of AT base pair with a twist angle of 36º, together with the individual contributions of the bases and the cross terms. Computed at ZORA-BLYP-D3(BJ)/TZ2P level of theory in gas on the geometries of the base pairs computed in water.[a]

		ΔE_{int}	ΔE_{Pauli}	ΔV_{elstat}	ΔE_{oi}	ΔE_{disp}
**DsPx/AT**	DsPx/AT	-14.9	23.1	-7.8	-4.4	-25.8
	Ds/A	-7.8	8.6	-4.4	-1.5	-10.5
	Px/T	-4.9	11.6	-3.9	-2.5	-10.2
	Px/A	-0.5	0.7	0.5	-0.3	-1.5
	Ds/T	-1.4	2.9	-0.1	-0.6	-3.6
**AT/AT**	AT/AT	-14.0	17.3	-5.8	-3.4	-22.1
	A/A	-6.0	7.9	-2.9	-1.3	-9.8
	T/T	-4.7	8.9	-1.8	-2.2	-9.6
	T/A	-1.6	0.7	-0.6	-0.2	-1.5
	A/T	-1.2	0.6	-0.3	-0.2	-1.2

[a] $\Delta E_{int} = \Delta E_{Pauli} + \Delta V_{elstat} + \Delta E_{oi} + \Delta E_{disp}$

Table S3. Cartesian coordinates of all optimized systems under analysis together with ADF total bonding energy (in kcal mol^{-1}, in parentheses). Computed at ZORA-BLYP-D3(BJ)/TZ2P in water.

A (-2320.6)

1	N	0.23077276	-2.04607889	0.00000000
2	C	-1.11329373	-1.93º163453	0.00000000
3	N	-1.85700261	-0.81026504	0.00000000
4	C	-1.08797806	0.29916673	0.00000000
5	C	0.31737462	0.33426964	0.00000000
6	C	0.98566771	-0.91317957	0.00000000
7	N	-1.47772198	1.62362844	0.00000000
8	C	-0.32827298	2.38639201	0.00000000
9	N	0.77683845	1.65173835	0.00000000
10	N	2.33210767	-1.02423479	0.00000000
11	H	-1.66325520	-2.87198567	0.00000000
12	H	-2.43052019	1.97286059	0.00000000
13	H	-0.36598847	3.46768363	0.00000000
14	H	2.91699147	-0.19941714	0.00000000
15	H	2.76428053	-1.93894378	0.00000000

T (-2182.2)

1	N	-1.20635001	1.30515619	0.00000000
2	C	-2.11204499	0.25785273	0.00000000
3	N	-1.50926530	-0.98467218	0.00000000
4	C	-0.14499320	-1.17081644	0.00000000
5	C	0.73491333	-0.13338845	0.00000000
6	C	0.19662769	1.21967501	0.00000000
7	O	0.87470391	2.26350099	0.00000000
8	O	-3.34106629	0.42059479	0.00000000
9	C	2.22685755	-0.32386442	0.00000000
10	H	-1.60690485	2.24151528	0.00000000
11	H	-2.12985027	-1.78804894	0.00000000
12	H	0.17463443	-2.20747135	0.00000000
13	H	2.68123179	0.14400986	0.88220363
14	H	2.48027437	-1.38805288	0.00000000
15	H	2.68123179	0.14400986	-0.88220363

G (-2484.8)

1	N	0.60317853	-2.84131954	0.00000000
2	O	2.63251345	1.33162816	0.00000000
3	N	1.53227184	-0.69925594	0.00000000

4	C	0.40827550	-1.50275226	0.00000000
5	N	-0.82975258	-1.00962723	0.00000000
6	C	-0.86573607	0.34435443	0.00000000
7	C	0.21502277	1.24269795	0.00000000
8	C	1.54410940	0.72043440	0.00000000
9	N	-1.98949275	1.13952095	0.00000000
10	C	-1.56379411	2.45714203	0.00000000
11	N	-0.24468377	2.56105138	0.00000000
12	H	1.52753913	-3.24975720	0.00000000
13	H	-0.20170294	-3.45351740	0.00000000
14	H	2.45060746	-1.13856333	0.00000000
15	H	-2.95217770	0.81811744	0.00000000
16	H	-2.26617811	3.27984619	0.00000000

C (-1923.6)

1	H	-1.22575050	2.71213843	0.00000000
2	O	-2.32336341	-1.87432143	0.00000000
3	C	-1.30236096	-1.14580842	0.00000000
4	N	-0.31238573	2.27550240	0.00000000
5	N	-1.35116365	0.21719946	0.00000000
6	C	1.08928138	0.31482506	0.00000000
7	N	-0.03465918	-1.75776077	0.00000000
8	C	1.12763393	-1.04654723	0.00000000
9	C	-0.20819731	0.92888162	0.00000000
10	H	1.99758067	0.90598396	0.00000000
11	H	-0.01344187	-2.77320306	0.00000000
12	H	2.04716746	-1.62095225	0.00000000
13	H	0.50965919	2.86406219	0.00000000

Ds (-3175.8)

1	C	2.96891276	0.97945346	0.00000000
2	C	-0.91706960	-1.09625320	0.00000000
3	C	-2.19943247	-3.25499369	0.00000000
4	H	2.99157325	3.11655203	0.00000000
5	C	-0.83519470	-3.44599409	0.00000000
6	H	-2.98637213	-3.99871527	0.00000000
7	C	-0.52891361	0.30681141	0.00000000
8	C	-0.10722148	-2.22420274	0.00000000
9	H	4.03551925	0.79528827	0.00000000
10	S	-2.61581534	-1.57411992	0.00000000

11	H	-0.37109954	-4.42756071	0.00000000
12	C	-1.47728659	1.35593370	0.00000000
13	C	0.81902856	0.74060279	0.00000000
14	H	0.97359072	-2.15317899	0.00000000
15	N	0.18525392	3.13750790	0.00000000
16	H	-1.86356351	3.47631707	0.00000000
17	N	2.45872641	2.25316247	0.00000000
18	C	-1.09740622	2.70327284	0.00000000
19	C	1.07736966	2.14133266	0.00000000
20	N	2.02862346	0.04145127	0.00000000
21	H	-2.53922283	1.12733280	0.00000000

Pa (-1728.7)

1	C	1.21062153	-1.13132778	0.00000000
2	C	0.57201986	1.04050153	0.00000000
3	N	-0.15006825	-1.08845587	0.00000000
4	C	-1.96203381	0.61112804	0.00000000
5	H	-0.77119937	-1.89007837	0.00000000
6	H	2.73646347	0.47112094	0.00000000
7	C	1.69283979	0.18420003	0.00000000
8	C	-0.58060978	0.23315703	0.00000000
9	H	-2.13809949	1.70499078	0.00000000
10	O	-2.92308509	-0.18482880	0.00000000
11	H	1.74113428	-2.07368124	0.00000000
12	H	0.57201688	2.12327375	0.00000000

Q (-2514.2)

1	C	1.14519388	-1.91935324	0.00000000
2	N	-1.56020555	1.16072583	0.00000000
3	C	-2.62919391	0.37337790	0.00000000
4	N	-2.31532943	-0.96488492	0.00000000
5	C	1.71149991	-0.63110558	0.00000000
6	C	0.91415715	0.52418765	0.00000000
7	C	-0.47012253	0.28465110	0.00000000
8	C	-0.93245736	-1.06004990	0.00000000
9	N	-0.18377019	-2.16896799	0.00000000
10	H	-2.97410668	-1.73650729	0.00000000
11	H	2.58076547	1.89163365	0.00000000
12	C	1.48703200	1.91557598	0.00000000
13	H	-3.65732188	0.71219952	0.00000000

14	H	1.79446845	-2.79317059	0.00000000
15	H	2.79503663	-0.53771181	0.00000000
16	H	1.14717704	2.47469984	-0.88133559
17	H	1.14717704	2.47469984	0.88133559

Pn (-1722.0)

1	C	5.70915610	-0.93340277	0.00000000
2	C	3.52471139	-0.31510308	0.00000000
3	N	4.95757112	-2.06602476	0.00000000
4	N	2.62068350	-2.69085934	0.00000000
5	H	5.29757828	-3.02175280	0.00000000
6	H	5.15090654	1.21026354	0.00000000
7	C	4.84347981	0.17317353	0.00000000
8	C	3.62147800	-1.71402756	0.00000000
9	O	1.41294371	-2.32295629	0.00000000
10	O	2.96667035	-3.90916656	0.00000000
11	H	6.78877801	-0.98177401	0.00000000
12	H	2.60440470	0.25092669	0.00000000

Px (-2448.5)

1	H	6.40362544	-3.46960942	0.00000000
2	H	4.94256706	-3.94798395	0.88737344
3	C	2.76092823	2.33275626	0.00000000
4	O	0.53845683	2.88598673	0.00000000
5	C	4.48124868	-0.87242155	0.00000000
6	C	4.85702862	-2.02751977	0.00000000
7	C	2.69765575	0.93794963	0.00000000
8	N	4.08748578	2.72175288	0.00000000
9	C	5.30790445	-3.41522410	0.00000000
10	O	2.05203269	4.51150789	0.00000000
11	H	5.95037617	1.69138769	0.00000000
12	H	4.94256706	-3.94798395	-0.88737344
13	C	4.03873017	0.47318848	0.00000000
14	H	1.79773583	0.34120972	0.00000000
15	C	4.87255374	1.61963649	0.00000000
16	N	1.73480744	3.28581162	0.00000000
17	H	4.39889803	3.68760770	0.00000000

AT (-4511.5)

1	N	-1.05924168	0.95457483	0.00000000

2	C	-2.40317020	1.05908141	0.00000000
3	N	-3.14526607	2.17876936	0.00000000
4	C	-2.37425477	3.28911574	0.00000000
5	C	-0.96898058	3.32751481	0.00000000
6	C	-0.29270495	2.08318100	0.00000000
7	N	-2.76694541	4.61237183	0.00000000
8	C	-1.61957861	5.37835035	0.00000000
9	N	-0.51314403	4.64555673	0.00000000
10	N	1.04732174	1.95678250	0.00000000
11	H	-2.94036992	0.11177001	0.00000000
12	H	-3.72064733	4.95922480	0.00000000
13	H	-1.65961526	6.45955904	0.00000000
14	H	1.62962177	2.78384816	0.00000000
15	H	1.47774926	1.02427735	0.00000000
16	N	0.08055162	-1.67859668	0.00000000
17	C	-0.81581628	-2.72796359	0.00000000
18	N	-0.21843637	-3.97716508	0.00000000
19	C	1.14353699	-4.16642331	0.00000000
20	C	2.02060464	-3.12572649	0.00000000
21	C	1.47201539	-1.77846513	0.00000000
22	O	2.17010540	-0.73699973	0.00000000
23	O	-2.04791163	-2.57147661	0.00000000
24	C	3.51313982	-3.31118135	0.00000000
25	H	-0.33677727	-0.71216404	0.00000000
26	H	-0.84207854	-4.77821030	0.00000000
27	H	1.46454191	-5.20277186	0.00000000
28	H	3.96706443	-2.84128979	0.88133248
29	H	3.77162140	-4.37425413	0.00000000
30	H	3.96706443	-2.84128979	-0.88133248

GC (-4421.7)

1	N	-1.85794345	-1.84157471	0.00000000
2	O	0.15529908	2.33226866	0.00000000
3	N	-0.93091672	0.29237669	0.00000000
4	C	-2.05789774	-0.50744324	0.00000000
5	N	-3.30340312	-0.01362427	0.00000000
6	C	-3.33853583	1.33751934	0.00000000
7	C	-2.25419498	2.23248383	0.00000000
8	C	-0.93266952	1.69820759	0.00000000
9	N	-4.46006513	2.13824925	0.00000000

10	C	-4.02852765	3.45475056	0.00000000
11	N	-2.70934737	3.55386477	0.00000000
12	H	-0.91605427	-2.26105345	0.00000000
13	H	-2.67239326	-2.44174493	0.00000000
14	H	0.00624818	-0.17163725	0.00000000
15	H	-5.42404045	1.82109583	0.00000000
16	H	-4.72766217	4.28036103	0.00000000
17	N	2.71986393	1.05307217	0.00000000
18	N	1.68895368	-1.01054839	0.00000000
19	C	4.12473990	-0.91020701	0.00000000
20	N	2.99767950	-2.98290632	0.00000000
21	C	4.16130436	-2.27044312	0.00000000
22	C	2.83194511	-0.28506523	0.00000000
23	H	5.03457587	-0.32144252	0.00000000
24	H	3.01713411	-3.99832260	0.00000000
25	H	5.07963517	-2.84643432	0.00000000
26	H	3.55247648	1.62789883	0.00000000
27	H	1.79311955	1.50625439	0.00000000
28	O	0.71185977	-3.09731144	0.00000000
29	C	1.73881707	-2.36864409	0.00000000

DsPa (-4905.8)

1	C	-1.48739069	-2.00609561	0.00000000
2	C	-1.00379866	-0.69330540	0.00000000
3	C	-1.36708104	1.79449023	0.00000000
4	C	-1.25536459	4.14320831	0.00000000
5	C	0.08865433	3.84234814	0.00000000
6	S	0.36462228	2.13305113	0.00000000
7	H	0.93401525	4.51876962	0.00000000
8	H	-0.78632132	-2.83849810	0.00000000
9	H	0.07190875	-0.54492053	0.00000000
10	O	2.79600814	-3.95910471	0.00000000
11	H	6.81369976	-0.92335094	0.00000000
12	N	-2.79979888	-2.33821551	0.00000000
13	C	-1.86601776	0.42748106	0.00000000
14	C	-2.08117830	2.98505708	0.00000000
15	H	-1.63796283	5.15926341	0.00000000
16	C	2.61099324	-2.72614785	0.00000000
17	C	-3.61098500	-1.27539781	0.00000000
18	C	-3.24369341	0.10106996	0.00000000

19	H	-3.16418726	3.00416695	0.00000000
20	C	3.64309658	-1.73484025	0.00000000
21	H	1.58395431	-2.31350114	0.00000000
22	N	-4.99663086	-1.27866231	0.00000000
23	N	-4.39458812	0.89262403	0.00000000
24	C	3.54095586	-0.33123023	0.00000000
25	N	4.99871915	-2.04243531	0.00000000
26	C	-5.40543063	0.03113633	0.00000000
27	H	-5.59524458	-2.09783159	0.00000000
28	C	4.85008706	0.19218080	0.00000000
29	H	2.61498275	0.22773102	0.00000000
30	C	5.73265441	-0.89604340	0.00000000
31	H	5.36822303	-2.98680184	0.00000000
32	H	-6.45430151	0.29826489	0.00000000
33	H	5.13740058	1.23553949	0.00000000

DsA (-5498.0)

1	N	-3.17898867	2.97714414	0.00000000
2	C	1.99945916	-1.92593744	0.00000000
3	N	-2.06003622	0.82461533	0.00000000
4	H	-1.13464510	2.67125551	0.00000000
5	C	2.08694216	-4.27793556	0.00000000
6	H	-0.06204686	-4.83941502	0.00000000
7	C	2.38509821	-0.52225579	0.00000000
8	C	2.81006417	-3.05301462	0.00000000
9	C	-4.32915966	2.27074882	0.00000000
10	C	-3.23231501	0.13430515	0.00000000
11	H	2.55537437	-5.25733426	0.00000000
12	C	1.43634912	0.52751042	0.00000000
13	C	3.73559667	-0.09519977	0.00000000
14	H	3.89055025	-2.97831788	0.00000000
15	C	-4.44165073	0.86904990	0.00000000
16	N	-5.63015870	2.73318116	0.00000000
17	N	-3.18973444	-1.21601475	0.00000000
18	C	1.82823322	1.87197224	0.00000000
19	C	4.00047094	1.30391610	0.00000000
20	N	4.94272294	-0.79909700	0.00000000
21	H	0.37011166	0.30901035	0.00000000
22	N	-5.78290846	0.48375143	0.00000000
23	C	-6.45544370	1.62777896	0.00000000

24	H	-5.92643369	3.70374718	0.00000000
25	H	-4.03947969	-1.76359287	0.00000000
26	H	-2.29378396	-1.68742696	0.00000000
27	N	3.11199350	2.30250339	0.00000000
28	H	1.07068305	2.65247907	0.00000000
29	N	5.38236544	1.41054720	0.00000000
30	C	5.88713710	0.13485751	0.00000000
31	H	-7.53302157	1.72540822	0.00000000
32	H	5.91858147	2.27182432	0.00000000
33	H	6.95293743	-0.05388116	0.00000000
34	S	0.30346503	-2.41340042	0.00000000
35	C	-2.10092930	2.17060369	0.00000000
36	C	0.72259992	-4.09338665	0.00000000

DsT (-5359.4)

1	C	-2.43438379	-2.36163937	0.00000000
2	N	-5.15044833	0.71627796	0.00000000
3	C	-6.21473333	-0.07851830	0.00000000
4	N	-5.89092847	-1.41170902	0.00000000
5	C	-1.86499339	-1.08283906	0.00000000
6	C	-2.65645051	0.08873731	0.00000000
7	C	-4.05271430	-0.14765781	0.00000000
8	C	-4.50753074	-1.49741380	0.00000000
9	N	-3.76567976	-2.60975086	0.00000000
10	H	-6.54110915	-2.19044519	0.00000000
11	C	-2.07567573	1.42291900	0.00000000
12	C	-2.71121853	2.65683691	0.00000000
13	C	-1.81510238	3.76172680	0.00000000
14	C	-0.49213643	3.38021638	0.00000000
15	S	-0.32757392	1.65736609	0.00000000
16	H	0.39307161	4.00340856	0.00000000
17	H	-2.13254187	4.79990091	0.00000000
18	H	-3.79105173	2.74168036	0.00000000
19	H	-7.24430798	0.25549341	0.00000000
20	H	-1.78483996	-3.23475667	0.00000000
21	H	-0.78046132	-1.01823377	0.00000000
22	N	2.65342353	-0.28492133	0.00000000
23	C	2.51315367	-1.66154302	0.00000000
24	N	3.72195922	-2.33169227	0.00000000
25	C	4.94472820	-1.69834863	0.00000000

26	C	5.06756164	-0.34339246	0.00000000
27	C	3.84851637	0.45317925	0.00000000
28	O	3.79879078	1.69681049	0.00000000
29	O	1.41308645	-2.23221063	0.00000000
30	C	6.39719420	0.35997579	0.00000000
31	H	1.78749769	0.25359449	0.00000000
32	H	3.67661939	-3.34580338	0.00000000
33	H	5.80282962	-2.36200735	0.00000000
34	H	6.49898272	1.00428067	-0.88226907
35	H	7.21748370	-0.36380218	0.00000000
36	H	6.49898272	1.00428067	0.88226907

DsG (-5659.7)

1	C	-1.18120864	-2.73380622	0.00000000
2	N	-5.10316746	-1.52145161	0.00000000
3	C	-5.59675350	-2.75442215	0.00000000
4	N	-4.62520372	-3.72347750	0.00000000
5	C	-1.36516219	-1.34726384	0.00000000
6	C	-2.64945167	-0.75567764	0.00000000
7	C	-3.71625764	-1.68630803	0.00000000
8	C	-3.40056422	-3.07562543	0.00000000
9	N	-2.18762944	-3.63856164	0.00000000
10	H	-4.77396748	-4.72710203	0.00000000
11	C	-2.84210269	0.68649379	0.00000000
12	C	-4.02755076	1.40946940	0.00000000
13	C	-3.83169296	2.81813612	0.00000000
14	C	-2.50067821	3.17346556	0.00000000
15	S	-1.46385750	1.78679485	0.00000000
16	H	-2.06567945	4.16501644	0.00000000
17	H	-4.64068727	3.54221515	0.00000000
18	H	-4.99587423	0.92417334	0.00000000
19	H	-6.64932622	-3.00654470	0.00000000
20	H	-0.17374131	-3.14631119	0.00000000
21	H	-0.48429470	-0.71206939	0.00000000
22	N	2.61455007	0.57644386	0.00000000
23	C	3.51604554	-0.46900621	0.00000000
24	N	4.83475034	-0.28104165	0.00000000
25	C	5.18822620	1.02692477	0.00000000
26	C	4.35028187	2.15522679	0.00000000
27	C	2.93487070	1.96303810	0.00000000

28	N	6.46804769	1.53388916	0.00000000
29	C	6.36593798	2.91466555	0.00000000
30	N	5.10873848	3.32756416	0.00000000
31	O	2.01817152	2.80682490	0.00000000
32	N	3.01392306	-1.72587851	0.00000000
33	H	1.61836831	0.37187748	0.00000000
34	H	7.32703371	0.99337247	0.00000000
35	H	7.24293143	3.54802244	0.00000000
36	H	3.65203392	-2.51031507	0.00000000
37	H	2.02094043	-1.90875156	0.00000000

DsC (-5100.4)

1	C	-1.39243007	-1.80833025	0.00000000
2	N	-4.43697195	0.94871417	0.00000000
3	C	-5.40592435	0.04021499	0.00000000
4	N	-4.93521228	-1.24851361	0.00000000
5	C	-0.97121330	-0.47303157	0.00000000
6	C	-1.88782227	0.60316329	0.00000000
7	C	-3.24908964	0.21249723	0.00000000
8	C	-3.55044024	-1.17961150	0.00000000
9	N	-2.68954700	-2.20265417	0.00000000
10	H	-5.49446109	-2.09503722	0.00000000
11	C	-1.45897497	1.99415311	0.00000000
12	C	-2.22865803	3.14920434	0.00000000
13	C	-1.46077245	4.34716926	0.00000000
14	C	-0.10381995	4.11427275	0.00000000
15	S	0.25235579	2.41959910	0.00000000
16	H	0.70765064	4.83084458	0.00000000
17	H	-1.89317924	5.34303819	0.00000000
18	H	-3.31126329	3.11458408	0.00000000
19	H	-6.46632349	0.25730349	0.00000000
20	H	-0.63862531	-2.59290337	0.00000000
21	H	0.10154882	-0.29561055	0.00000000
22	N	2.52765207	-1.30896073	0.00000000
23	C	2.60562352	-2.67119828	0.00000000
24	N	3.89004572	-3.25515030	0.00000000
25	C	5.03660382	-2.51990623	0.00000000
26	C	4.96786746	-1.15972884	0.00000000
27	C	3.65610410	-0.57592837	0.00000000
28	N	3.52445976	0.76915489	0.00000000

29	O	1.60642567	-3.42707245	0.00000000
30	H	3.93257779	-4.26991527	0.00000000
31	H	5.96845927	-3.07431626	0.00000000
32	H	5.86242956	-0.54788158	0.00000000
33	H	2.60132367	1.18672715	0.00000000
34	H	4.33360113	1.37510983	0.00000000

DsPn (-4899.4)

1	C	-1.84682066	-2.12494852	0.00000000
2	C	-1.25210445	-0.85803458	0.00000000
3	C	-1.40842694	1.65183701	0.00000000
4	C	-1.10349551	3.98398159	0.00000000
5	C	0.21101723	3.57351390	0.00000000
6	S	0.34486116	1.84703059	0.00000000
7	H	1.10921242	4.17811575	0.00000000
8	H	-1.21470162	-3.01062675	0.00000000
9	H	-0.16736380	-0.81028921	0.00000000
10	O	3.38941613	-3.88715430	0.00000000
11	H	6.98616603	-0.68299635	0.00000000
12	N	-3.18305869	-2.34562686	0.00000000
13	C	-2.01867328	0.33041815	0.00000000
14	C	-2.02159435	2.89739019	0.00000000
15	H	-1.40094017	5.02816277	0.00000000
16	N	2.95470926	-2.69681085	0.00000000
17	C	-3.90165629	-1.21818669	0.00000000
18	C	-3.41936056	0.12219960	0.00000000
19	H	-3.09942252	3.00442549	0.00000000
20	C	3.88282732	-1.64982000	0.00000000
21	O	1.72462030	-2.41595090	0.00000000
22	N	-5.28302125	-1.10440412	0.00000000
23	N	-4.49975138	1.00812246	0.00000000
24	C	3.68133352	-0.26287734	0.00000000
25	N	5.24105290	-1.90163803	0.00000000
26	C	-5.57982509	0.23512680	0.00000000
27	H	-5.94884660	-1.86990880	0.00000000
28	C	4.96004507	0.32298467	0.00000000
29	H	2.72152503	0.23108754	0.00000000
30	C	5.90596368	-0.71529728	0.00000000
31	H	5.65031752	-2.82981840	0.00000000
32	H	-6.60250115	0.58977020	0.00000000

33	H	5.18849674	1.38022234	0.00000000

DsPx (-5626.9)

1	C	-2.37265137	2.41711854	0.00000000
2	C	-1.70445000	1.18753068	0.00000000
3	C	-1.70441058	-1.32886728	0.00000000
4	C	-1.26109528	-3.63770999	0.00000000
5	C	0.02626004	-3.14891483	0.00000000
6	S	0.05788662	-1.41774216	0.00000000
7	H	0.95532149	-3.70263282	0.00000000
8	H	-1.79408481	3.33821699	0.00000000
9	H	-0.61973700	1.20714473	0.00000000
10	O	2.83587608	5.02075074	0.00000000
11	H	5.86833347	1.26917530	0.00000000
12	N	-3.71958146	2.55518572	0.00000000
13	C	-2.39395510	-0.04668134	0.00000000
14	C	-2.24287445	-2.60850525	0.00000000
15	H	-1.49448874	-4.69796376	0.00000000
16	N	2.21536756	3.91736839	0.00000000
17	C	-4.36776513	1.38567556	0.00000000
18	C	-3.80472203	0.07690205	0.00000000
19	H	-3.31230584	-2.78027986	0.00000000
20	C	2.96218991	2.73232700	0.00000000
21	O	0.95692548	3.83311097	0.00000000
22	N	-5.73936674	1.18777731	0.00000000
23	N	-4.82872567	-0.87305285	0.00000000
24	C	2.53457422	1.40485614	0.00000000
25	N	4.34433274	2.75714465	0.00000000
26	C	-5.95397271	-0.16734394	0.00000000
27	H	-6.45053609	1.91138128	0.00000000
28	C	3.70267043	0.60073102	0.00000000
29	H	1.51173037	1.06784619	0.00000000
30	C	4.81009043	1.48552162	0.00000000
31	H	4.90139656	3.60539220	0.00000000
32	H	-6.95304959	-0.58374097	0.00000000
33	H	3.35901508	-3.89801097	0.88851996
34	C	3.76511540	-0.81456188	0.00000000
35	C	3.80561128	-2.02820598	0.00000000
36	C	3.85452966	-3.48571171	0.00000000
37	H	3.35901508	-3.89801097	-0.88851996

38	H	4.89153067	-3.84322056	0.00000000

QPa (-4243.5)

1	C	-1.39895476	-1.96636548	0.00000000
2	N	-4.45917522	0.76110842	0.00000000
3	C	-5.42399682	-0.15095897	0.00000000
4	N	-4.94902755	-1.44091045	0.00000000
5	C	-0.99592826	-0.61895579	0.00000000
6	C	-1.92596770	0.43158636	0.00000000
7	C	-3.27065977	0.02486298	0.00000000
8	C	-3.56510523	-1.36656970	0.00000000
9	N	-2.68720591	-2.37634645	0.00000000
10	H	-6.48576628	0.05977777	0.00000000
11	C	-1.52615161	1.88194382	0.00000000
12	H	-0.43845314	1.99357254	0.00000000
13	H	-1.93040620	2.39545975	0.88134993
14	H	-5.50861773	-2.28726663	0.00000000
15	H	-0.64789572	-2.75448781	0.00000000
16	H	0.06743074	-0.39729108	0.00000000
17	H	-1.93040620	2.39545975	-0.88134993
18	C	2.94129365	0.95294457	0.00000000
19	C	3.63500151	-0.27196784	0.00000000
20	N	4.99134404	0.03256131	0.00000000
21	C	5.16273727	1.38271491	0.00000000
22	C	3.89870776	1.98742118	0.00000000
23	C	3.13110668	-1.61192197	0.00000000
24	H	6.15091376	1.82187179	0.00000000
25	H	5.73078638	-0.66144838	0.00000000
26	O	3.83223686	-2.64296171	0.00000000
27	H	2.02586361	-1.68638740	0.00000000
28	H	1.86484160	1.05927459	0.00000000
29	H	3.71145426	3.05327990	0.00000000

QA (-4835.5)

1	N	-3.08612160	2.35275397	0.00000000
2	H	2.33396378	-2.93409052	-0.88128918
3	N	-1.83704771	0.27398868	0.00000000
4	H	-1.02428001	2.17314619	0.00000000
5	C	6.06403139	-0.74265245	0.00000000
6	H	-7.35386610	0.83268073	0.00000000

7	C	2.52482486	-0.97778565	0.00000000
8	H	6.35770114	1.37494508	0.00000000
9	C	-4.19005118	1.57591466	0.00000000
10	C	-2.96292030	-0.48820770	0.00000000
11	H	7.10012004	-1.05638687	0.00000000
12	C	1.69712850	0.15610939	0.00000000
13	C	3.90313811	-0.70623017	0.00000000
14	C	-1.95999053	1.61538919	0.00000000
15	C	-4.21559888	0.16973263	0.00000000
16	N	-5.51723820	1.95664691	0.00000000
17	N	-2.83702206	-1.83394901	0.00000000
18	C	2.23486481	1.45644616	0.00000000
19	C	4.33279568	0.64925566	0.00000000
20	N	5.01434125	-1.55571138	0.00000000
21	H	0.61545648	0.03665162	0.00000000
22	N	-5.53023147	-0.29798132	0.00000000
23	C	-6.27230945	0.80218038	0.00000000
24	H	-5.87302547	2.90697651	0.00000000
25	H	-3.65191258	-2.43229904	0.00000000
26	H	-1.91644161	-2.25380307	0.00000000
27	N	3.55753878	1.73973979	0.00000000
28	H	1.56693229	2.31634191	0.00000000
29	N	5.71788959	0.58755976	0.00000000
30	C	1.98346352	-2.38219025	0.00000000
31	H	2.33396378	-2.93409052	0.88128918
32	H	0.88990311	-2.38108127	0.00000000

QT (-4696.6)

1	C	-1.78848727	-0.89573658	0.00000000
2	N	-5.37304561	1.09819647	0.00000000
3	C	-6.11506178	-0.00276156	0.00000000
4	N	-5.36960556	-1.15768616	0.00000000
5	C	-1.69149306	0.50783991	0.00000000
6	C	-2.82919903	1.32995305	0.00000000
7	C	-4.05222704	0.63935484	0.00000000
8	C	-4.03523553	-0.78266461	0.00000000
9	N	-2.95785084	-1.57576718	0.00000000
10	H	-5.73035941	-2.10597785	0.00000000
11	H	-1.71681841	3.17617653	0.00000000
12	C	5.76723472	0.77077344	0.00000000

13	H	1.25945738	-0.18631333	0.00000000
14	H	3.77829254	-3.37350790	0.00000000
15	H	5.68503463	-2.01352021	0.00000000
16	H	5.74847918	1.42263520	-0.88232292
17	H	6.70677979	0.21048221	0.00000000
18	H	5.74847918	1.42263520	0.88232292
19	H	-7.19724358	-0.02930533	0.00000000
20	H	-0.88557697	-1.50444770	0.00000000
21	H	-0.70652587	0.97055104	0.00000000
22	N	2.20728509	-0.55471756	0.00000000
23	C	2.32317361	-1.93460466	0.00000000
24	N	3.63457597	-2.36856271	0.00000000
25	C	4.71911127	-1.52003742	0.00000000
26	C	4.59009010	-0.16565401	0.00000000
27	C	3.24568334	0.39282522	0.00000000
28	O	2.96693738	1.60530368	0.00000000
29	O	1.34780531	-2.69847907	0.00000000
30	C	-2.75506221	2.83255273	0.00000000
31	H	-3.26231361	3.24523214	-0.88114654
32	H	-3.26231361	3.24523214	0.88114654

QG (-4998.0)

1	C	1.11060133	-2.46390306	0.00000000
2	N	-2.87549097	-1.51300568	0.00000000
3	C	-3.29266046	-2.77344490	0.00000000
4	N	-2.26312330	-3.68427854	0.00000000
5	C	0.82789052	-1.08685910	0.00000000
6	C	-0.48929990	-0.60263908	0.00000000
7	C	-1.48029613	-1.59807804	0.00000000
8	C	-1.08015362	-2.96291909	0.00000000
9	N	0.17161946	-3.43518424	0.00000000
10	H	-2.35462744	-4.69475804	0.00000000
11	H	0.08484922	1.47483413	0.00000000
12	O	4.17778531	2.86839206	0.00000000
13	N	5.13862359	-1.67082879	0.00000000
14	H	3.75967861	0.43392786	0.00000000
15	H	9.47260585	1.01788232	0.00000000
16	H	9.40487092	3.57311403	0.00000000
17	H	5.76911599	-2.46149404	0.00000000
18	H	4.14404351	-1.84416647	0.00000000

19	H	-4.32746667	-3.09117913	0.00000000
20	H	2.14283063	-2.81048844	0.00000000
21	H	1.65193974	-0.37803520	0.00000000
22	N	4.75715671	0.63330246	0.00000000
23	C	5.65152662	-0.41868798	0.00000000
24	N	6.97147730	-0.23996881	0.00000000
25	C	7.33416515	1.06525369	0.00000000
26	C	6.50364421	2.19926236	0.00000000
27	C	5.08784700	2.01537265	0.00000000
28	N	8.61710203	1.56388934	0.00000000
29	C	8.52386392	2.94535968	0.00000000
30	N	7.26947598	3.36654109	0.00000000
31	C	-0.82236578	0.86418633	0.00000000
32	H	-1.42197310	1.12471952	-0.88118802
33	H	-1.42197310	1.12471952	0.88118802

QC (-4437.5)

1	C	-0.22346427	-2.89803746	0.00000000
2	N	-3.24067519	-0.11916560	0.00000000
3	C	-4.22078310	-1.01486136	0.00000000
4	N	-3.76706321	-2.31236283	0.00000000
5	C	0.20224330	-1.55641953	0.00000000
6	C	-0.71149727	-0.49174657	0.00000000
7	C	-2.06353154	-0.87541934	0.00000000
8	C	-2.38118543	-2.26140641	0.00000000
9	N	-1.52040490	-3.28626394	0.00000000
10	H	-4.34074054	-3.14916785	0.00000000
11	H	0.80302381	1.04268980	0.00000000
12	N	4.66868739	-0.31517544	0.00000000
13	O	2.77848504	-4.52058565	0.00000000
14	H	5.11268248	-5.35203001	0.00000000
15	H	7.14035066	-4.14300008	0.00000000
16	H	7.02008016	-1.61794406	0.00000000
17	H	3.74080287	0.08946749	0.00000000
18	H	5.46983358	0.30126623	0.00000000
19	H	-5.27893524	-0.78647094	0.00000000
20	H	0.52729282	-3.68620856	0.00000000
21	H	1.27350682	-1.36937373	0.00000000
22	N	3.69121072	-2.40002692	0.00000000
23	C	3.77498259	-3.76128941	0.00000000

24	N	5.06325746	-4.33754658	0.00000000
25	C	6.20489659	-3.59469012	0.00000000
26	C	6.12877777	-2.23446533	0.00000000
27	C	4.81349904	-1.65967572	0.00000000
28	C	-0.28734669	0.95199381	0.00000000
29	H	-0.68231793	1.47405383	-0.88124767
30	H	-0.68231793	1.47405383	0.88124767
